# Ceramide-mediated gut dysbiosis enhances cholesterol esterification and promotes colorectal tumorigenesis in mice

**DOI:** 10.1172/jci.insight.150607

**Published:** 2022-02-08

**Authors:** Yahui Zhu, Li Gu, Xi Lin, Jinmiao Zhang, Yi Tang, Xinyi Zhou, Bingjun Lu, Xingrong Lin, Cheng Liu, Edward V. Prochownik, Youjun Li

**Affiliations:** 1 Frontier Science Center for Immunology and Metabolism, Hubei Key Laboratory of Cell Homeostasis, College of Life Sciences, and; 2 Medical Research Institute, Wuhan University, Wuhan, China.; 3 Division of Hematology/Oncology, Children’s Hospital of Pittsburgh of University of Pittsburgh Medical Center (UPMC), Department of Microbiology and Molecular Genetics, Pittsburgh Liver Research Center and Hillman Cancer Center of UPMC, UPMC, Pittsburgh, Pennsylvania, USA.

**Keywords:** Gastroenterology, Metabolism, Cholesterol, Colorectal cancer, Oncogenes

## Abstract

Colorectal cancer (CRC) severely threatens human health and life span. An effective therapeutic strategy has not been established because we do not clearly know its pathogenesis. Here, we report that ceramide and sterol *O*-acyltransferase 1 (SOAT1) have roles in both spontaneous and chemical-induced intestinal cancers. We first found that miRNA-148a deficiency dramatically increased mouse gut dysbiosis through upregulating ceramide synthase 5 (Cers5) expression, which promoted ceramide synthesis afterward. The newly generated ceramide further promoted both azoxymethane/dextran sodium sulfate–induced (AOM/DSS-induced) and *Apc^Min/+^* spontaneous intestinal tumorigenesis via increasing mouse gut dysbiosis. Meanwhile, increased level of ceramide correlated with the significant enhancements of both β-catenin activity and colorectal tumorigenesis in a TLR4-dependent fashion. Next, we found a direct binding of β-catenin to *SOAT1* promoter to activate transcriptional expression of *SOAT1*, which further induced cholesterol esterification and colorectal tumorigenesis. In human patients with CRC, the same CERS5/TLR4/**β**-catenin/SOAT1 axis was also found to be dysregulated. Finally, the SOAT1 inhibitor (avasimibe) showed significant levels of therapeutic effects on both AOM/DSS-induced and *Apc^Min/+^* spontaneous intestinal cancer. Our study clarified that ceramide promoted CRC development through increasing gut dysbiosis, further resulting in the increase of cholesterol esterification in a SOAT1-dependent way. Treatment with avasimibe to specifically decrease cholesterol esterification could be considered as a clinical strategy for effective CRC therapy in a future study.

## Graphical abstract

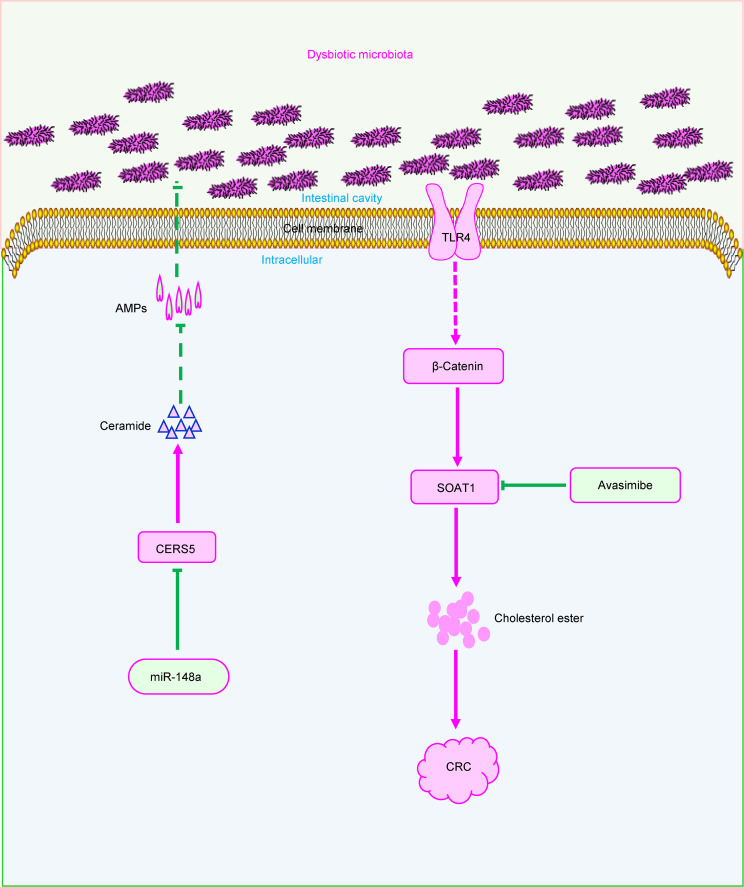

## Introduction

Colorectal cancer (CRC) threatens human health and life span, which is evidenced by findings that both incidence and mortality are within the top 3 positions among all clinically diagnosed tumors (1, 2). Although many factors have been found to induce CRC, including genetic factors, diet, living habits, dysregulation of gut microbes, and obesity (3–5), the specific molecular mechanism underlying these effects to finally cause CRC has not been uncovered yet. Moreover, there is still no clear molecular target for treatment of CRC.

Gut microbiota was found to be implicated in colon tumorigenesis (6–9). In these reports, both genetic and environmental factors affected the composition of the gut microbiota. Gut microbiota imbalance also altered host metabolism and immune signals to regulate colon tumorigenesis (10–13). Accumulating evidence showed that colonic bacteria, such as Clostridia, *Bacteroides*, and *Bifidobacterium*, was involved in colitis and colitis-associated tumorigenesis in studies with both experimental mouse models and patients in the clinic (14–19). Previous studies also showed that altered colonic bacterial composition affected the susceptibility to colitis and colitis-associated cancers in several gene knockout (KO) animal models, such as *Nlrp6*, *Asc*, *Il18*, *Aim2*, and *Il-33* KO mouse models (8, 13, 16, 19, 20). Besides findings in these animal models, particularly, miRNA-148a (miR-148a) was originally identified as the most obvious tumor suppressor in many cancers, such as CRC, liver cancer, and gastric cancer (21–24). Remarkably, our previous results demonstrated that the miR-148a–deficient mice were highly susceptible to intestinal inflammation and colitis-associated tumorigenesis because of the significant inhibitions on critical regulators for both NF-κB and STAT3 signaling in a proinflammatory environment (22). After this finding, however, we are still unsure whether and how miR-148a regulates gut microbiota imbalance to contribute to colorectal tumorigenesis.

Ceramide synthase 5 (CERS5) was previously found to be responsible for ceramide synthesis, as well as to contribute to diet-induced obesity and hepatic triglyceride accumulation in mice (25–27). Wegner et al. demonstrated that CERS5 was upregulated in estrogen receptor–positive cancer tissues (28). Meanwhile, a recent study indicated that high expression of CERS5 correlated with poor survival rate of patients with CRC (29). Moreover, CERS5 had high expression levels in most CRC tissues relative to paracancer tissues (30, 31). However, whether CERS5 regulates gut microbiota balance and how the expression of CERS5 is regulated in CRC are still poorly understood.

Toll-like receptor 4 (TLR4), as one of the conserved innate immune sensors, is activated by microbial structures (32). TLR4 activation promotes colorectal tumorigenesis through induction of NF-κB and inflammation (22, 32, 33). Moreover, TLR4 also induces cholesterol synthesis and colorectal tumorigenesis via enhancing SREBP2 activity in mice (34). Meanwhile, many of the cancer-causing bacteria in the gut promote intestinal tumorigenesis by inducing hyperactivation of the TLR4 signaling pathway (34–36). However, whether ceramide induces alterations in the gut microbiota to specifically regulate TLR4 activity and whether TLR4 regulates cholesterol ester synthesis have not been analyzed.

Sterol *O*-acyltransferase 1 (SOAT1) is known to contribute to the growths of liver cancer, pancreatic cancer, and prostate cancer through promoting cholesterol ester synthesis (37, 38). Cholesterol ester increases hepatocellular carcinoma growth through promoting synthesis of phospholipids and hormones, both as the raw materials for cell membrane synthesis and as signaling molecules (39–41). However, whether SOAT1 contributes to CRC development through inducing cholesterol ester synthesis is still unknown.

## Results

### Loss of miR-148a increases gut dysbiosis to enhance colorectal tumorigenesis.

**Figure 1:**
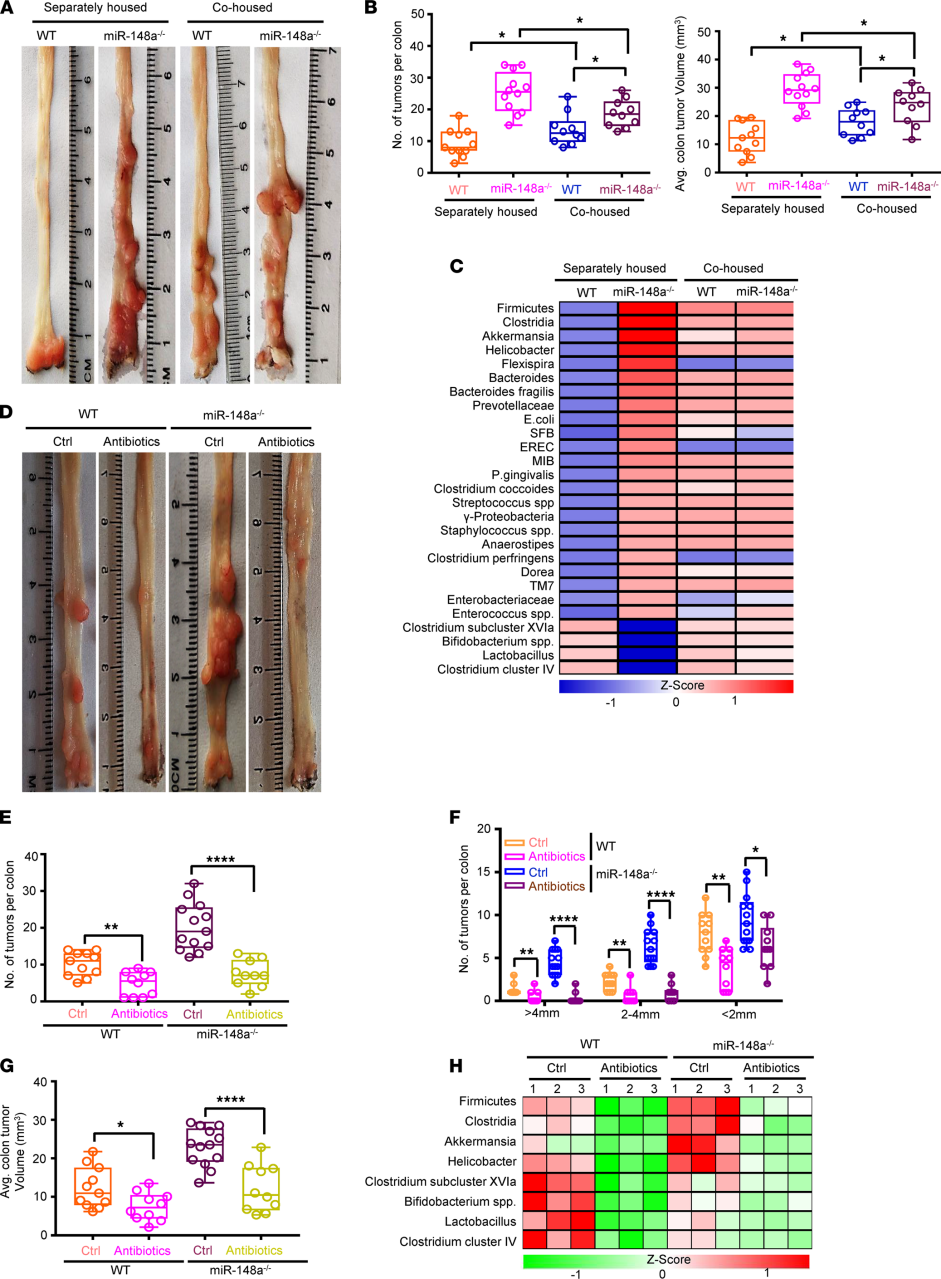
Depletion of miR-148a increases gut dysbiosis to enhance colorectal tumorigenesis. (**A**) Representative images of colon tumors from WT and *miR-148a^–/–^* mice under separately housed and cohoused situations. (**B**) Colon tumor numbers (left) and average tumor volumes (right) from **A** (*n* = 10–12/group). (**C**) The relative levels of indicated gut microbiota of mice from **A** after quantitative PCR (qPCR) analysis (*n* = 3/group). (**A**–**C**) Separately housed or cohoused WT and *miR-148a^–/–^* mice were injected with AOM on day 0 and were treated with 3 rounds of 2.5% DSS in drinking water from day 0 to day 7 for 7 days followed by regular drinking water. (**D**) Representative images of colon tumors from WT and *miR-148a^–/–^* mice treated with control (Ctrl) or antibiotics mix. (**E**–**G**) Colon tumor numbers (**E**), tumor sizes, (**F**) and average tumor volumes (**G**) from **D** (*n* = 10–13/group). (**H**) qPCR analyses on bacterial 16s rDNA of gut microbiota from **D** (*n* = 3/group). (Data were presented as mean ± SEM in **B**, and **E**–**G**.) *P < 0.05, **P < 0.01, ****P < 0.0001. Statistical significance was calculated by using 1-way ANOVA (**B**) or 2-tailed unpaired *t* test (**E**, **F**, and **G**).

To investigate whether miR-148a loss regulates gut microbiota disorders in azoxymethane/dextran sodium sulfate–induced (AOM/DSS-induced) CRC, cohousing experiments were performed to find that the susceptibility to colitis-associated tumorigenesis was able to be transferred to the cohoused normal mice by equilibrating the gut microbiota (7–9, 13, 42). Compared with the separately housed *miR-148a^–/–^* mice, we found that the cohoused *miR-148a^–/–^* mice with WT mice exhibited significantly decreased weights (Supplemental Figure 1A; supplemental material available online with this article; https://doi.org/10.1172/jci.insight.150607DS1), as well as numbers and sizes of colon tumors (Figure 1, A and B, and Supplemental Figure 1B). On the other hand, the cohoused WT mice exhibited the opposite changes compared with the separately housed WT ones (Figure 1, A and B; and Supplemental Figure 1, A and B). Together, these results suggested that gut microbiota imbalance played a critical role in colorectal tumorigenesis in *miR-148a^–/–^* mice. Next, we analyzed the difference of gut microbiota composition among the separately housed mice, as well as the cohoused WT and *miR-148a^–/–^* ones after AOM/DSS treatments through using 16s ribosomal DNA (rDNA) analysis. As shown in Figure 1C, there was a notably distinct gut microbiota composition when comparing the separately housed WT and *miR-148a^–/–^* mice while gut microbiota composition showed almost no difference in these 2 types of mice when they were cohoused after induction of CRC. Compared with the separately housed WT mice, the separately housed *miR-148a^–/–^* ones exhibited increased levels of Firmicutes, Clostridia, *Akkermansia*, and *Helicobacter* (Figure 1C). On the other hand, they exhibited decreased levels of *Bifidobacterium* and *Lactobacillus* species (Figure 1C). Meanwhile, the above gut bacteria remained at almost stable levels during the times when these mice were cohoused (Figure 1C). To find out the reason that the KO of miR-148a–induced alterations in the microbiota caused CRC, instead of that CRC caused alterations in the gut microbiota, the levels of gut microbiota composition were detected in the untreated littermates of both WT and *miR-148a^–/–^* mice. Results showed that depletion of *miR-148a* significantly increased levels of gut dysbiosis in these mice compared with the levels in separately housed littermates of WT mice under the condition of no treatment (Supplemental Figure 1C). Meanwhile, endotoxin levels also significantly increased in the separately housed *miR-148a^–/–^* compared with WT mice (Supplemental Figure 1D). However, the endotoxin remained at almost stable levels when these mice were cohoused (Supplemental Figure 1D). The depletion of *miR-148a* also induced elevated levels of proinflammatory cytokines compared with WT in the separately housed mice with no treatment or after AOM/DSS treatment (Supplemental Figure 1, E and F). However, cohoused mice could partially rescue proinflammatory cytokines that were increased due to *miR-148a* deficiency in mice with no treatment and after AOM/DSS treatment (Supplemental Figure 1, E and F). Results indicated that *miR-148a^–/–^* mice induced increases in tumor numbers, volumes, and diameters compared with the separately housed littermates of WT mice (Supplemental Figure 1, G and H). Compared with the separately housed *miR-148a^–/–^* mice, the *miR-148a^–/–^* mice cohoused with littermates of WT mice exhibited decreased levels of both numbers and sizes of colon tumors (Supplemental Figure 1, G and H) while the cohoused WT mice exhibited the opposite changes in both levels compared with the separately housed WT ones (Supplemental Figure 1, G and H). These effects were mainly caused by the expanded bacteria in the *miR-148a^–/–^* mice that were transmitted to the cohoused littermate WT mice. Meanwhile, depletion of miR-148a significantly increased levels of gut dysbiosis in *miR-148a^–/–^* mice compared with littermates of WT mice under the separately housed condition (Supplemental Figure 1I). The levels of composition of gut microbiota were similar in cohoused littermates of both *miR-148a^–/–^* and WT mice (Supplemental Figure 1I). Together, the above data suggested that the altered microbial community induced by miR-148a deficiency could be a major factor in colorectal cancer development.

To further determine whether the increases in various gut bacteria played some important roles in *miR-148a^–/–^* mice, we treated these mice with mixed antibiotics for 4 months (Supplemental Figure 1J). Treatments with antibiotics significantly repressed colon tumors as shown by tumor numbers, volumes, and diameters in WT and *miR-148a^–/–^* mice (Figure 1, D–G). Remarkably, the inhibitory effects of antibiotics were found to be more effective in *miR-48a^–/–^* mice than in WT mice (Figure 1, D–G). Finally, treatments with antibiotics significantly inhibited the growths of Firmicutes, Clostridia, *Akkermansia*, and *Helicobacter* in *miR-148a^–/–^* mice (Figure 1H). It was found that the endotoxin concentration decreased significantly while the levels of mRNAs for proinflammatory cytokines decreased partially in *miR-148a^–/–^* mice treated with antibiotics (Supplemental Figure 1, K and L). In WT mice, however, both endotoxin concentration and the levels of mRNAs for proinflammatory cytokines decreased slightly (Supplemental Figure 1, K and L). Taken together, these data demonstrated that, in *miR-148a^–/–^* mice, colorectal tumorigenesis was significantly promoted, which correlated with enhanced levels of Firmicutes, Clostridia, *Akkermansia*, and *Helicobacter*.

### miR-148a directly inhibits CERS5 expression via binding to its 3′UTR.

**Figure 2:**
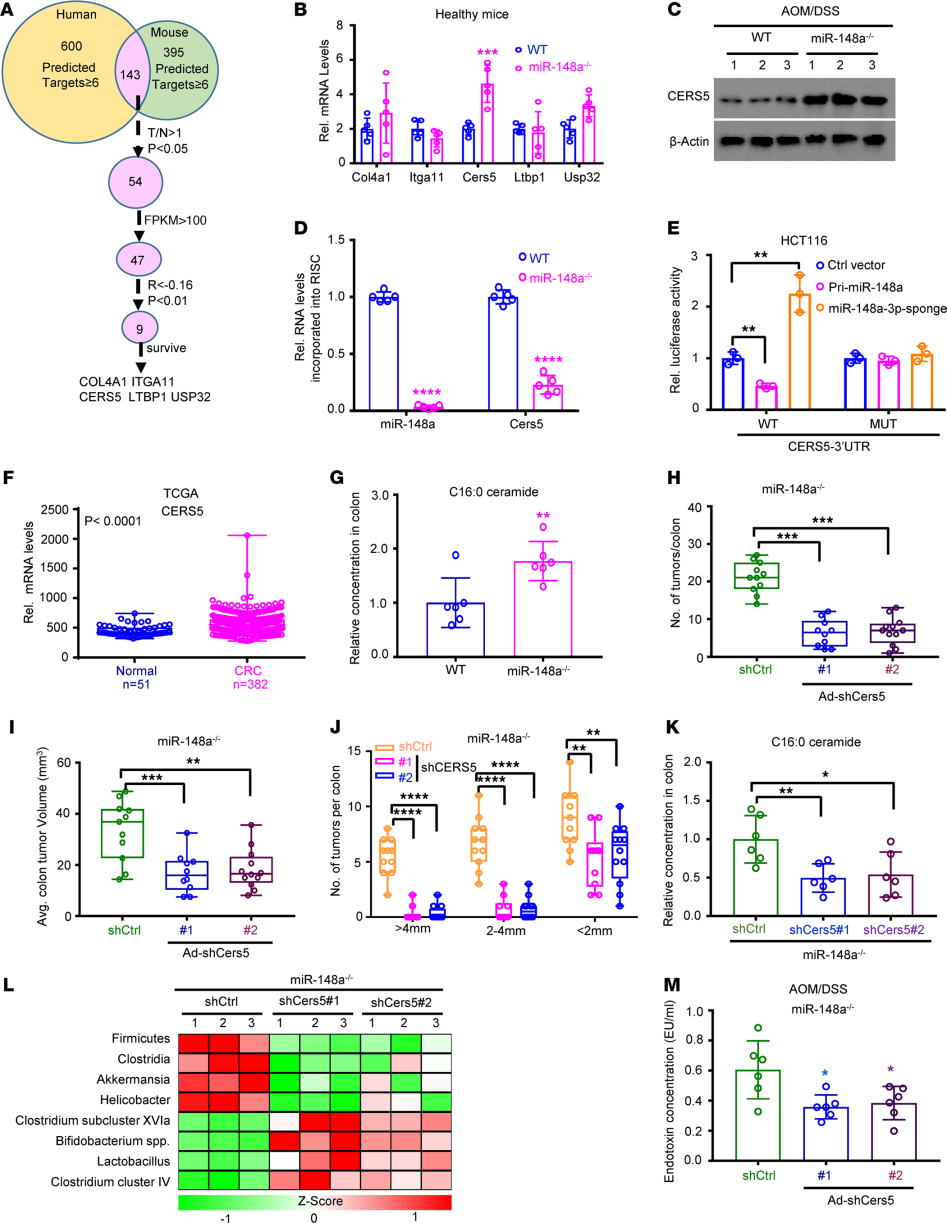
Depletion of miR-148a upregulates CERS5 to increase colorectal carcinogenesis. (**A**) Summary of bioinformatics screening results in CRCs. (**B**) qPCR analyses on the mRNA levels of *Col4a1*, *Itga11*, *Cers5*, *Ltbp1*, and *Usp32* in WT and *miR-148a^–/–^* mice colon tissues (*n* = 5/group). (**C**) Western blot assay obtained the CERS5 protein levels in CRC from indicated mice (*n* = 3/group). (**D**) Reverse transcription qPCR (RT-qPCR) assay was performed to quantitate the targeted mRNAs of miR-148a-3p that incorporated into the RISC derived from colon tissues of either WT or *miR-148a^–/–^* mice. β-Actin was used as a control (*n* = 5/group). (**E**) Luciferase activity of the reporter vector containing WT or miR-148a-3p binding mutant 3′UTR of miR-148a-3p targets was determined in the HCT116 CRC cells transfected with indicated vectors. (**F**) *CERS5* expression in normal colon and CRC tissues from TCGA data sets. (**G**) The colon C16:0 ceramide was detected by using mass spectrometry in WT mice and *miR-148a^–/–^* mice (*n* = 5/group). (**H**–**J**) Colon tumor numbers (**H**), average volumes, (**I**) and sizes (**J**) in indicated mice (*n* = 10–12/group). (**K**) The colon C16:1 ceramide was detected by using mass spectrometry in CRCs of indicated mice (*n* = 6/group). (**L**) qPCR analyses on the indicated microbiota in mice from **H** (*n* = 3/group). (**M**) The endotoxin concentrations in *miR-148a^–/–^* mice from **H** (*n* = 6/group). (Data were presented as mean ± SEM in **B**, **D**–**K**, and **M**.) *P < 0.05, **P < 0.01, ***P < 0.001, ****P < 0.0001. Statistical significance was calculated by using 1-way ANOVA (**E** and **H**–**K**) or 2-tailed unpaired *t* test (**B**, **D**, **F**, **G**, and **M**). Data shown in **E** are representatives of 3 independent experiments. T/N, tumor/normal; FPKM, fragments per kilobase million; EU, endotoxin units.

Next, we investigated the mechanism of how miR-148a regulates microbial communities. We searched the target genes of miR-148a in both humans and mice through multiple websites. A total of 143 common target genes were found in more than 6 websites (Figure 2A). Meanwhile, 54 genes among them were found to have higher levels of expression in the tumors than normal tissues in The Cancer Genome Atlas (TCGA) CRC database (Figure 2A). Interestingly, 9 target genes were found to negatively correlate with miR-148a expression in TCGA CRC database (Figure 2A). Remarkably, 5 genes (*COL4A1*, *ITGA11*, *CERS5*, *LTBP1*, and *USP32*) were found to have upregulated expressions in CRC patients with poor prognosis (Figure 2A). We further detected their expressions in WT and *miR-148a^–/–^* mouse colon tissues. Results indicated that only *Cers5* mRNA was significantly upregulated in the colon tissues of *miR-148a^–/–^* mice without treatment with AOM/DSS (Figure 2B). Meanwhile, *CERS5* 3′UTR was found to contain a miR-148a-3p binding site (Supplemental Figure 2A). Our results also indicated that CERS5 protein level increased significantly in both normal colons and CRC of *miR-148a^–/–^* mice (Figure 2C and Supplemental Figure 2B). In addition, ectopic miR-148a expression significantly inhibited CERS5 protein levels in HCT116 and SW480 cells (Supplemental Figure 2C). Similar results were found in WT mouse colon tissues that had induced miR-148a expression after administration of lentivirus that carried miR-148a–expressing vector (Supplemental Figure 2, D and E). Furthermore, restoring miR-148a expression in *miR-148a^–/–^* mice prevented the increase of CERS5 (Supplemental Figure 2, F and G). In order to further study whether CERS5 is a direct miR-148a-3p target, IP with an antibody against argonaute RISC catalytic component 2 (AGO2) was used to precipitate the RNAs associated with the RNA-induced silencing complex (RISC). As shown in Figure 2D, the *Cers5* mRNA was more enriched in the extracts from colon tissues of WT mice than those from colon tissues of *miR-148a^–/–^* mice. Moreover, luciferase reporter assays were conducted to further determine whether the putative miR-148a-3p binding sites at the 3′UTR for its targets are critical for miR-148a-3p–mediated inhibition. As expected, ectopic miR-148a expression inhibited the activity of human 3*′*UTR reporter vector in dual luciferase reporter assays, while mutation in miR-148a-3p binding sites abrogated this inhibition (Figure 2E and Supplemental Figure 2H). On the other hand, repression of miR-148a-3p using its sponge vector increased the activity of 3*′*UTR reporter vectors, while mutation in the miR-148a-3p binding site abrogated this upregulation (Figure 2E and Supplemental Figure 2H). Taken together, these data suggested that miR-148a-3p directly inhibited CERS5 expression through binding to its 3*′*UTR.

Next, we analyzed the mRNA levels of *CERS5* in both CRC tissues and normal para-CRC tissues from samples of TCGA CRC patients. As shown in Figure 2F, *CERS5* was significantly overexpressed in CRC samples compared with normal ones. Meanwhile, results of qPCR also indicated that mRNA levels of *CERS5* were highly expressed in CRC samples compared with normal tissues adjacent to CRC (Supplemental Figure 2I). Results of detection of CERS5 protein levels also revealed that CERS5 level was significantly higher in tumor than nontumor after AOM/DSS induction (Supplemental Figure 2J). Because CERS5 is known to mainly contribute to the synthesis of C16:0 ceramide (25, 43), the content of C16:0 ceramide was detected through mass spectrometry in CRCs of WT and *miR-148a^–/–^* mice. The results showed that the concentration of C16:0 ceramide was robustly increased in *miR-148a^–/–^* CRCs versus WT ones (Figure 2G). Taken together, the identified CERS5 might play an extremely critical role during CRC development, especially under the condition of miR-148a deficiency.

### Depletion of Cers5 represses colorectal tumorigenesis through ceramide in miR-148a^–/–^ mice.

To further study the role of CERS5 in CRC in *miR-148a*–deficient mice, we investigated whether depletion of its expression would inhibit tumor growth in CRC mouse models. Concentrated adenovirus carrying a *Cers5* shRNA vector was administrated to AOM/DSS-treated *miR-148a^–/–^* mice (Supplemental Figure 2K), and tumor burden was assessed after mice were sacrificed on day 120 of administration (Supplemental Figure 2K). Results of Western blot showed that CERS5 protein was suppressed in miR-*148a^–/–^* mice administrated with adenovirus carrying a *Cers5* shRNA (Supplemental Figure 2L). Next, we found that depletion of *Cers5* could significantly repress the colon tumor as shown by tumor numbers, tumor volumes, and tumor diameters in *miR-148a^–/–^* mice (Figure 2, H–J). Meanwhile, the lipid metabolomics results indicated that depletion of *Cers5* could significantly decrease CRC C16:0 ceramide levels in *miR-148a^–/–^* mice (Figure 2K). Finally, the gut microbiota levels were determined in the gut of these mice. Results showed that the levels of potentially carcinogenic bacteria were significantly lower in *Cers5* deletion mice guts than in control mice (Figure 2L). The depletion of *Cers5* also significantly reduced endotoxin concentration and inflammation in *miR-148a^–/–^* mice before and after AOM/DSS induction (Figure 2M and Supplemental Figure 2, M and N). Taken together, the above data suggested that depletion of *miR-148a* increased C16:0 ceramide synthesis and gut dysbiosis through upregulating CERS5 expression in mice.

### Ceramide promotes CRC growth via increasing gut dysbiosis.

**Figure 3:**
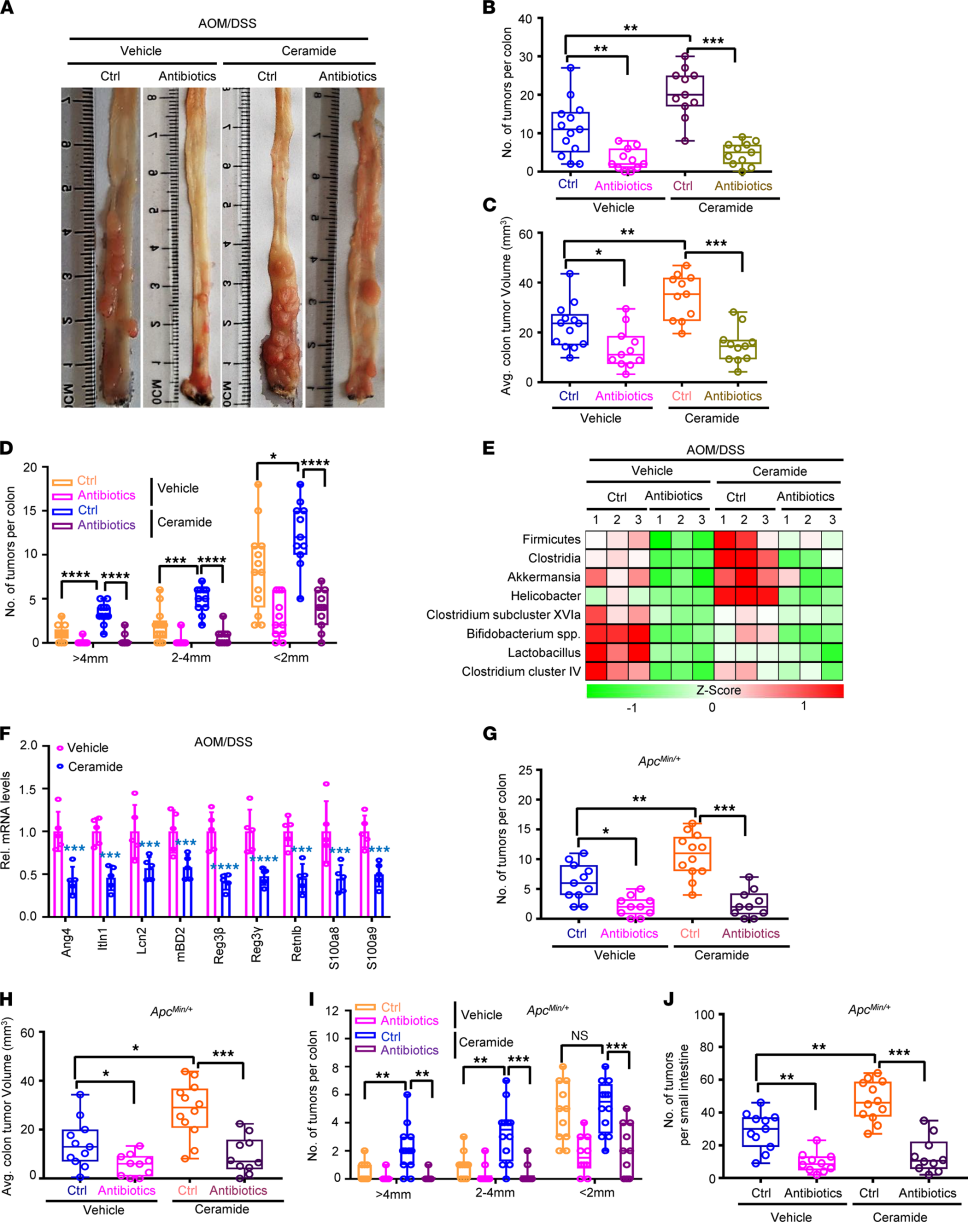
Ceramide increases gut dysbiosis to promote colorectal tumorigenesis. (**A**) Representative images of colon tumor from AOM/DSS-induced mice with indicated treatment. (**B**–**D**) The colon tumor numbers (**B**), average tumor volumes (**C**) and tumor sizes (**D**) from **A** (*n* = 11–13/group). (**E**) qPCR analyses on bacterial 16s rDNA in indicated gut microbiota from **A** (*n* = 3/group). (**F**) The relative mRNA levels of AMPs for mice from **A** by qPCR analysis (*n* = 5/group). (**G**–**I**) Colon tumor numbers (**G**), average tumor volumes, (**H**) and tumor sizes (**I**) from indicated mice (*n* = 10–12/group). (**J**) The small intestine tumor numbers of indicated mice (*n* = 10–12/group). (Data were presented as mean ± SEM in **B**–**D** and **F**–**J**.) *P < 0.05, **P < 0.01, ***P < 0.001, ****P < 0.0001. Statistical significance was calculated by using 1-way ANOVA (**B**–**D** and **G**–**J**) or 2-tailed unpaired *t* test (**F**).

The data from above experiments suggested that the absence of miR-148a could induce the production of C16:0 ceramide. Next, we investigated whether C16:0 ceramide affected colorectal tumorigenesis through inducing gut microbe composition imbalance. To assess the role of ceramide in CRC development, we injected C16:0 ceramide into mice treated with AOM/DSS by intraperitoneal injection (Supplemental Figure 3A). To investigate whether C16:0 ceramide induces alterations in the gut microbiota in AOM/DSS-induced CRC, we used antibiotics treatment in these mice (Supplemental Figure 3A). The tumor burden was assessed after the mice were sacrificed on day 120 of treatment (Supplemental Figure 3A). Remarkably, results showed that C16:0 ceramide could significantly increase colon tumor numbers, volumes, and diameters in AOM/DSS-induced CRC (Figure 3, A–D). Meanwhile, the results also revealed that antibiotics could significantly repress the colon tumor growth induced by C16:0 ceramide (Figure 3, A–D). Results of qPCR indicated that C16:0 ceramide could significantly promote alteration of the gut microbial community in these mice before and after AOM/DSS induction (Supplemental Figure 3B and Figure 3E). Moreover, antibiotics treatment could significantly inhibit gut dysbiosis in C16:0 ceramide-treated WT mice before and after AOM/DSS induction (Supplemental Figure 3B and Figure 3E). As mucus and antimicrobial peptides (AMPs) are critical for regulating gut microbiota composition, we investigated whether C16:0 ceramide affects gut microbial communities through regulating mucus and AMPs’ expression in colon of WT mice. Our data indicated that C16:0 ceramide significantly decreased mucus and AMPs’ expressions in the colons of WT mice before and after AOM/DSS induction (Figure 3F and Supplemental Figure 3, C–E). These data indicated that C16:0 ceramide promoted CRC development through inducing alterations in the gut microbiota in AOM/DSS-induced CRC.

We next analyzed the role of ceramide in a spontaneous intestinal cancer model of *Apc^Min/+^* mice (Supplemental Figure 3F). Results showed that C16:0 ceramide could significantly accelerate spontaneous colon tumor numbers, volumes, and diameters in *Apc^Min/+^* mice (Figure 3, G–I). Meanwhile, the C16:0 ceramide also could significantly boost spontaneous small intestine tumor numbers, volumes, and diameters in *Apc^Min/+^* mice (Figure 3J and Supplemental Figure 3G). Moreover, antibiotics treatment could decrease spontaneous colon and small intestine tumor numbers, volumes, and diameters induced by ceramide in *Apc^Min/+^* mice (Figure 3, G–J, and Supplemental Figure 3G). The above data indicated that C16:0 ceramide could significantly promote AOM/DSS-induced and spontaneous intestinal cancer dependent on altered gut microbial community.

### Ceramide-mediated alterations in the gut microbiota boost colorectal tumorigenesis dependent on TLR4.

**Figure 4:**
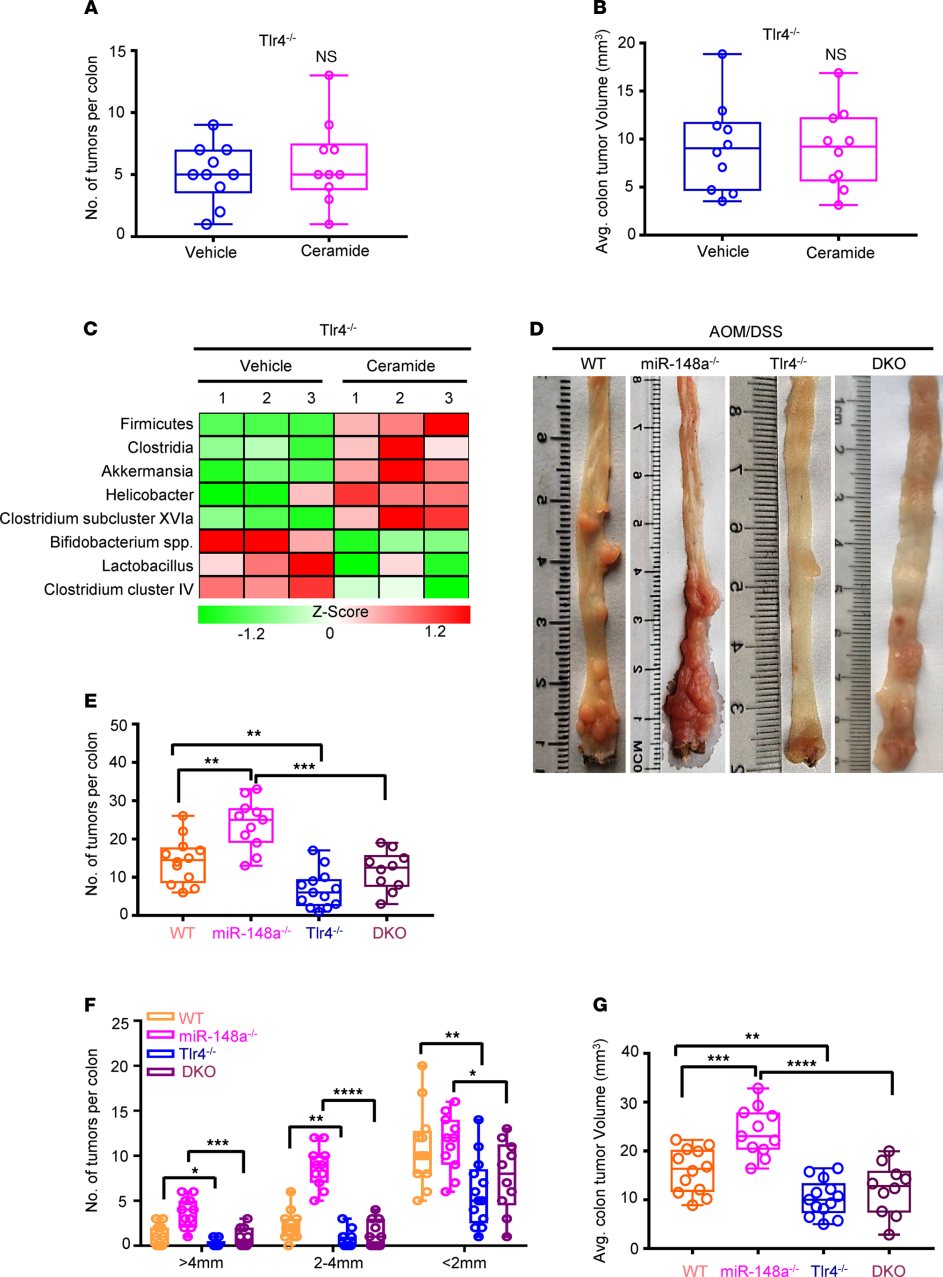
miR-148a depletion promotes CRC growth dependent on ceramide-mediated alterations in the gut microbiota to activate TLR4. (**A** and **B**) Colon tumor numbers (**A**) and average tumor volumes (**B**) from Supplemental Figure 4A (*n* = 10/group). (**C**) qPCR analyses on bacterial 16s rDNA in indicated gut microbiota from Supplemental Figure 4A (*n* = 3/group). (**D**) Representative images of colon tumor induced by AOM/DSS from WT, *miR-148a^–/–^*, *Tlr4^–/–^*, and *miR-148a^–/–^ Tlr4^–/–^* mice. (**E**–**G**) The colon tumor numbers (**E**), tumor sizes, (**F**) and average tumor volumes (**G**) in indicated mice from **D** (*n* = 10–13/group). (Data were presented as mean ± SEM in **A**, **B**, and **E**–**G**.) *P < 0.05, **P < 0.01, ***P < 0.001, ****P < 0.0001. Statistical significance was calculated by using 1-way ANOVA (**E**–**G**) or 2-tailed unpaired *t* test (**A** and **B**).

Previous studies revealed that the functions of many gut microorganisms are dependent on TLR4 (42, 44). Here, we further investigated whether ceramide-mediated alterations in the gut microbiota promoted colorectal tumorigenesis dependent on TLR4 in mice. First, we treated *Tlr4^–/–^* mice using C16:0 ceramide by intraperitoneal injection. Consistent with the previous results, C16:0 ceramide could not increase colon tumor numbers and volumes in *Tlr4^–/–^* mice (Figure 4, A and B; and Supplemental Figure 4, A and B). qPCR analysis results showed that C16:0 ceramide treatment could induce alterations in the gut microbiota in *Tlr4^–/–^* mice (Figure 4C). Together, all these results indicated that C16:0 ceramide promotes colorectal tumorigenesis dependent on TLR4 in vivo.

### Tlr4 loss attenuates colorectal tumorigenesis driven by miR-148a deficiency.

To study whether *Tlr4* played important roles in colorectal tumorigenesis in *miR-148a^–/–^* mice, we crossed *Tlr4^–/–^* mice with *miR-148a^–/–^* mice to generate *miR-148a^–/–^ Tlr4^–/–^* mice and analyzed CRC development following AOM/DSS treatment. Our results showed that depletion of *Tlr4* decreased colon tumor numbers, volumes, and diameters compared with WT mice (Figure 4, D–G). Remarkably, depletion of *Tlr4* could also significantly inhibit colon tumor numbers, volumes, and diameters induced by miR-148a deletion (Figure 4, D–G). These results suggested that TLR4 played some critical role on miR-148a^–/–^ CRC.

### Ceramide-mediated alterations in the gut microbiota increased SOAT1 expression through TLR4.

**Figure 5:**
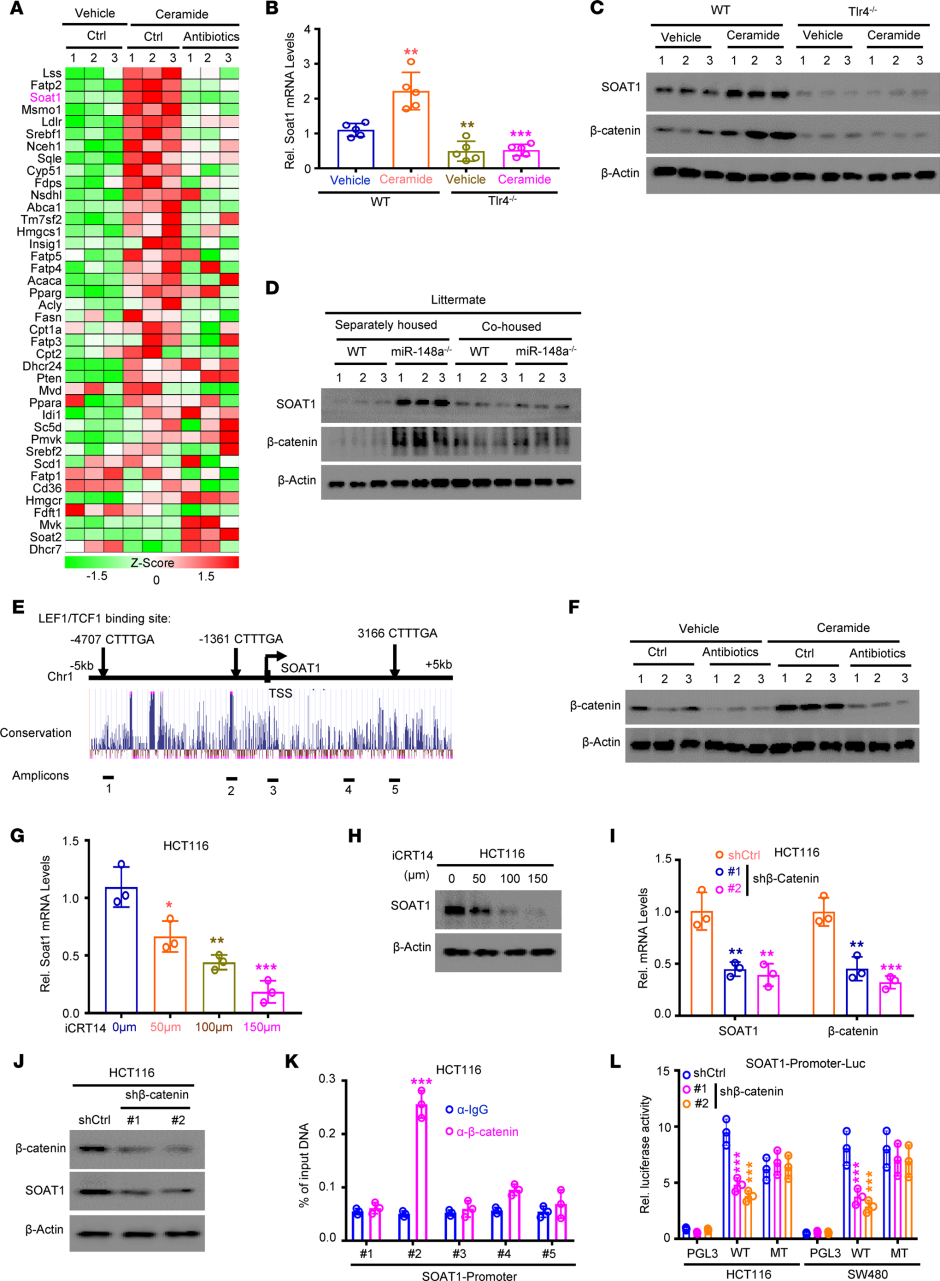
SOAT1 is transcriptionally activated by β-catenin/TCF1 complex. (**A**) qPCR analyzed lipid metabolism genes’ expression on CRCs from AOM/DSS-treated vehicle + control, ceramide + control, and ceramide + antibiotics mice (*n* = 3/group). (**B** and **C**) The mRNA (**B**) (*n* = 5/group) and protein (**C**) (*n* = 3/group) levels of SOAT1 in CRCs of WT and *Tlr4^–/–^* mice with indicated treatment. (**D**) Results of Western blot assay for both β-catenin and SOAT1 protein levels in CRC from indicated mice (*n* = 3/group). (**E**) Schematic representation of the *SOAT1* promoter (–5 kb to +5 kb of the transcription start site [TSS]). At the top, β-catenin binding sites are indicated. (**F**) Western blot detected β-catenin protein levels in CRCs of mouse with indicated treatment (*n* = 3/group). (**G** and **H**) The mRNA (**G**) and protein (**H**) levels of SOAT1 in HCT116 cells treated with different concentrations of β-catenin inhibitor (iCRT14). (**I** and **J**) Depletion of β-catenin repressed the mRNA (**I**) and protein (**J**) levels of SOAT1 in HCT116 cells. (**K**) ChIP qPCR analyses on the *SOAT1* promoter with IgG and β-catenin antibodies in HCT116 cells. (**L**) Luciferase activity of *SOAT1* promoter in HCT116 and SW480 cells in response to shRNA-mediated suppression of β-catenin. (Data were presented as mean ± SEM in **B**, **G**, **I**, **K**, and **L**.) *P < 0.05, **P < 0.01, ***P < 0.001. Statistical significance was calculated by using 1-way ANOVA (**B**) or 2-tailed unpaired *t* test (**G**, **I**, **K**, and **L**). Data shown in **G**–**L** are representatives of 3 independent experiments.

To analyze lipid metabolism gene expression regulated by C16:0 ceramide-induced alterations in the gut microbiota, gene expression levels were compared in various CRCs from vehicle + control (Ctrl), ceramide + Ctrl, and ceramide + antibiotics WT mice. Remarkably, *Soat1*, which catalyzes the formation of fatty acid cholesterol esters, was one of the most highly upregulated genes in ceramide-treated WT mice (Figure 5A). Meanwhile, *Soat1* expression decreased in antibiotics-treated mice compared with control mice (Figure 5A). Moreover, ceramide had no effect on *Soat1* expression in antibiotics-treated mice (Figure 5A). Next, we studied whether C16:0 ceramide regulated SOAT1 expression through TLR4 in mice. Results revealed that C16:0 ceramide also significantly promoted expressions of both SOAT1 mRNA and protein in WT mice instead of *Tlr4^–/–^* mice (Figure 5, B and C). Depletion of *miR-148a* could induce alterations in the gut microbiota through inducing ceramide synthesis. Next, we decided to detect SOAT1 expression in *miR-148a^–/–^* mice. Results indicated that SOAT1 was substantially upregulated in the separately housed *miR-148a^–/–^* mice rather than in the cohoused compared with littermate WT mice (Figure 5D). These data indicated that ceramide-mediated microbial community alterations could induce SOAT1 expression dependent on TLR4 in CRC.

### SOAT1 is transcriptionally activated by β-catenin/T cell factor 1 complex.

Next, we investigated through which signaling pathway SOAT1 expression was induced in ceramide-mediated microbial community alteration to induce CRC. Toward this aim, we analyzed the putative transcription factor binding sites within the *SOAT1* promoter regions (–5 to +5 kb) in the MotifMap website. Remarkably, T cell factor 1/lymphoid enhancer-binding factor 1 (TCF1/LEF1) emerged as the top candidate, and its candidate binding sites were largely conserved in *SOAT1* promoter (Figure 5E). We next investigated whether C16:0 ceramide-mediated alterations in the gut microbiota regulate β-catenin expression. C16:0 ceramide could substantially increase β-catenin protein level, and antibiotics treatment could repress β-catenin expression induced by C16:0 ceramide in mouse CRCs (Figure 5F). Meanwhile, C16:0 ceramide also led to increased activity of β-catenin through its nuclear localization in mouse CRCs (Supplemental Figure 5A). In addition, antibiotics could rescue the β-catenin activity induced by C16:0 ceramide (Supplemental Figure 5A). Meanwhile, *Tlr4* depletion could substantially reduce β-catenin protein levels induced by C16:0 ceramide in mouse CRCs (Figure 5C). Results of Western blot also suggested that β-catenin protein levels dramatically increased in *miR-148a^–/–^* mice when compared with littermate WT mice in a separately housed environment rather than cohoused environment (Figure 5D). Our previous data suggested that alterations in the gut microbiota induced by miR-148a deficiency may play a critical role in CRC. To study the function of alterations in the microbiota induced by miR-148a deficiency in CRC, mixed gut bacteria were isolated from the gut of WT or *miR-48a^–/–^* mice induced by AOM/DSS. Next, the gut microbiota mixes were used to treat HCT116 cells, then to analyze the β-catenin expression. Results showed that gut microbiota mixes from *miR-148a^–/–^* mice significantly increased β-catenin levels dependent on TLR4 in HCT116 cells (Supplemental Figure 5B). Meanwhile, the gut microbiota mixes from *miR-148a^–/–^* mice, instead of WT mice, promoted cancer cell growth dependent on TLR4 (Supplemental Figure 5C). LPS treatment of TLR4 ligand also significantly enhanced both β-catenin activity and SOAT1 expression in control HCT116 cells compared with in the HCT116 cells with stable knockdown of TLR4 (Supplemental Figure 5D). These data suggested that C16:0 ceramide-mediated alterations in the gut microbiota induced β-catenin activation through TLR4 in CRCs. Next, we analyzed whether WNT/β-catenin signaling was directly responsible for SOAT1 expression in CRC. Particularly, we characterized the relationship between activation of WNT/β-catenin and expressions of SOAT1 in HCT116 and SW480 cells. Our results indicated that the levels of both SOAT1 mRNA and protein were highly downregulated following treatment of CRC cells with iCRT14, a potent inhibitor of the β-catenin and TCF/LEF interaction (Figure 5, G and H; and Supplemental Figure 5, E and F). Depletion of β-catenin had a similar effect (Figure 5, I and J; and Supplemental Figure 5, G and H). Meanwhile, the mRNA and protein levels of SOAT1 were dramatically increased in HCT116 and SW480 cells with overexpression of β-catenin (Supplemental Figure 5, I and J).

We next tested whether β-catenin directly transactivated SOAT1, using ChIP assays. Our results showed that β-catenin occupied the promoters of SOAT1 in HCT116 and SW480 cells (Figure 5K and Supplemental Figure 5K). To confirm this finding, luciferase reporters containing SOAT1 promoter elements were used to confirm the responsive ectopic β-catenin expression (Supplemental Figure 5L). Moreover, shRNA-mediated β-catenin suppression dramatically decreased luciferase activity driven by WT promoters, but not by those with mutant β-catenin binding sites (Figure 5L). Thus, C16:0 ceramide altered the community diversity in the gut and induced β-catenin activation to facilitate colorectal tumorigenesis via direct upregulation of SOAT1 in CRC.

### SOAT1 mediates colorectal tumorigenesis driven by miR-148a deficiency.

**Figure 6:**
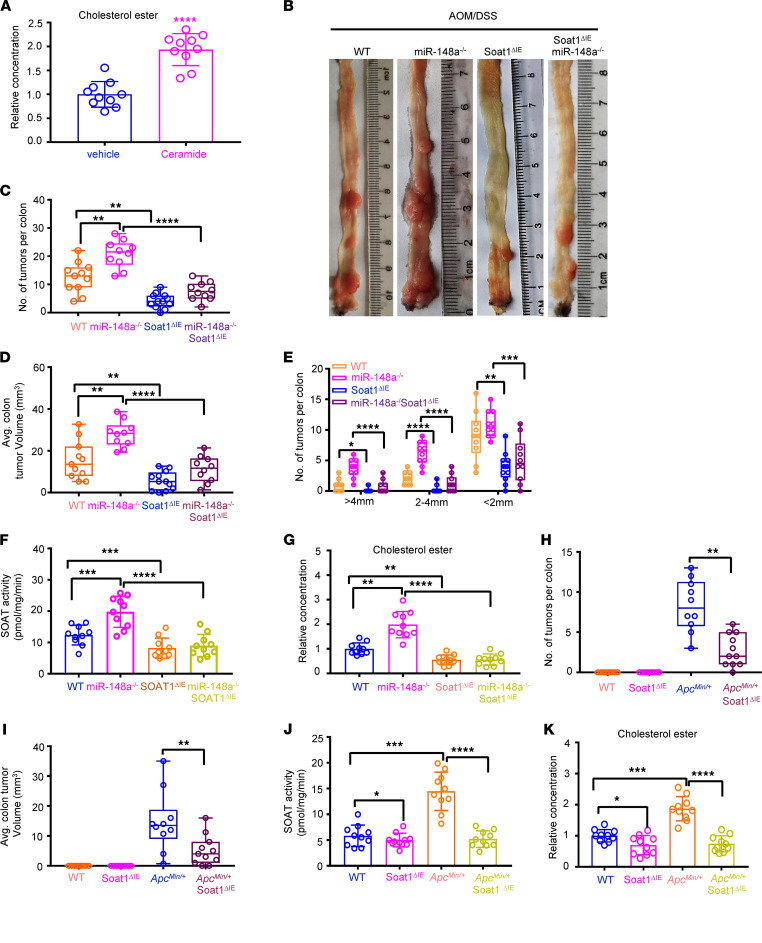
Soat1 loss attenuates intestinal carcinogenesis in *miR-148a^–/–^* and *Apc^Min/+^* mice. (**A**) The CRC cholesterol ester levels from WT mice with indicated treatments (*n* = 10/group). (**B**) Representative images of colon tumors induced by AOM/DSS from WT, *miR-148a^–/–^*, *Soat1*^ΔIE^, and *miR-148a^–/–^ Soat1*^ΔIE^ mice. (**C**–**E**) The colon tumor numbers (**C**), average tumor volumes, (**D**) and tumor sizes (**E**) in the indicated mice from **B** (*n* = 10–12/group). (**F**) The SOAT1 activity in CRCs from **B** (*n* = 10/group). (**G**) The colon cholesterol ester levels from indicated mice (*n* = 10/group). (**H** and **I**) The colon tumor numbers (**H**) and average tumor volumes (**I**) in indicated mice (*n* = 10–11/group). (**J** and **K**) The SOAT1 activity (**J**) and cholesterol ester (**K**) levels in CRCs from indicated mice (*n* = 10/group). (Data were presented as mean ± SEM in **A** and **C**–**K**.) *P < 0.05, **P < 0.01, ***P < 0.001, ****P < 0.0001. Statistical significance was calculated by using 1-way ANOVA (**C**–**G**, **J**, and **K**) or 2-tailed unpaired *t* test (**A**, **H**, and **I**).

The above data showed that ceramide induced SOAT1 expression through β-catenin. Meanwhile, ceramide significantly boosted SOAT1 activity in CRC (Supplemental Figure 6A). Since SOAT1 was mainly responsible for regulating the production of cholesterol esters, it was necessary to clarify the details of how cholesterol esters changed in *miR-148a^–/–^* mice. Remarkably, these data also indicated that total cholesterol and cholesterol ester robustly increased in the separately housed *miR-148^–/–^* mice when compared with those in the littermate WT mice, but all changes did not happen in the cohoused mice (Supplemental Figure 6, B and C). After our specially designed detections, results indicated that it was ceramide that increased cholesterol ester synthesis significantly in these mice (Figure 6A).

Our above results also showed that miR-148a deficiency accelerated colorectal tumorigenesis through a CERS5/ceramide/TLR4/β-catenin axis. Because the ceramide/TLR4/β-catenin axis regulated SOAT1 expression in CRC, the role of SOAT1 in ceramide-mediated colorectal tumorigenesis was investigated by us. In order to study the function of SOAT1 in vivo, we generated we generated mice with intestinal epithelial cell–specific knockout of SOAT1 (*Soat1^fl/fl^ Villin-Cre*; hereafter *Soat1*^ΔIE^). The role of C16:0 ceramide in colorectal tumorigenesis of *Soat1*^ΔIE^ mice was assessed afterward. Results indicated that C16:0 ceramide could not promote colorectal tumorigenesis in *Soat1*^ΔIE^ mice (Supplemental Figure 6, D–F). However, ceramide could still induce the alterations in the gut microbiota of these mice (Supplemental Figure 6G). Taken together, these data indicated that SOAT1 was necessary for C16:0 ceramide to promote colorectal tumorigenesis.

To investigate the function of SOAT1 on colorectal tumorigenesis induced in *miR-148a^–/–^* mice, we then crossed *Soat1*^ΔIE^ mice with *miR-148a^–/–^* mice to generate miR-*148a^–/–^ Soat1*^ΔIE^ mice for inducing CRC with AOM/DSS. It was found that the numbers, volumes, and diameters of colon tumors in *Soat1*^ΔIE^ mice were all significantly lower than those in control mice (Figure 6, B–E), which was consistent with our above findings. *Soat1* KO also significantly decreased these properties in *miR-148a^–/–^* mice (Figure 6, B–E). In addition, basal activity of SOAT1 was also increased in *miR-148a^–/–^* tumors when compared with WT tumors (Figure 6F). Furthermore, our data also showed that both total cholesterol and cholesterol ester significantly increased in *miR-148a^–/–^* tumors but significantly decreased in *Soat1*^ΔIE^ tumors (Figure 6G and Supplemental Figure 6H). Together, all these results suggested that SOAT1 promoted colorectal tumorigenesis in *miR-148a^–/–^* mice via inducing cholesterol ester synthesis.

### Depletion of Soat1 attenuates spontaneous intestinal tumor development in Apc^Min/+^ mice.

Previous reports revealed that *Apc^Min/+^* mice genetically induced intestinal and colon tumors through inducing excessive activation of β-catenin (45, 46). Thus, we detected the SOAT1 protein levels in *Apc^Min/+^* mice to characterize the possible interaction between SOAT1 and *Apc* mutant induced spontaneous intestinal cancer. Our results showed that SOAT1 protein levels were significantly upregulated in *Apc^Min/+^* mice when compared with WT ones (Supplemental Figure 6I). To investigate how *Soat1* deficiency affected spontaneous tumor development, *Soat1*^ΔIE^ mice were crossed with *Apc^Min/+^* mice to observe the details of tumor development. Results revealed that depletion of *Soat1* significantly repressed both volumes and sizes of tumors, as well as tumor multiplicities for both colon and small intestine tumors in *Apc^Min/+^* mice (Figure 6, H and I; and Supplemental Figure 6, J and K) when compared with WT littermates on an *Apc^Min/+^* mouse background. Meanwhile, the levels of SOAT1 activity, total cholesterol, and cholesterol ester were all significantly increased in *Apc^Min/+^* mice (Figure 6, J and K, and Supplemental Figure 6L). On the other hand, *Soat1* depletion significantly induced the decreases on levels of total cholesterol and cholesterol ester through repressing SOAT1 activity in *Apc^Min/+^* mice (Figure 6, J and K, and Supplemental Figure 6L). All together, these data suggested that depletion of *Soat1* attenuated spontaneous colon and small intestine tumor development of *Apc^Min/+^* mice.

### The CERS5/ceramide/β-catenin/SOAT1 signaling axis was dysregulated in human CRC samples.

**Figure 7:**
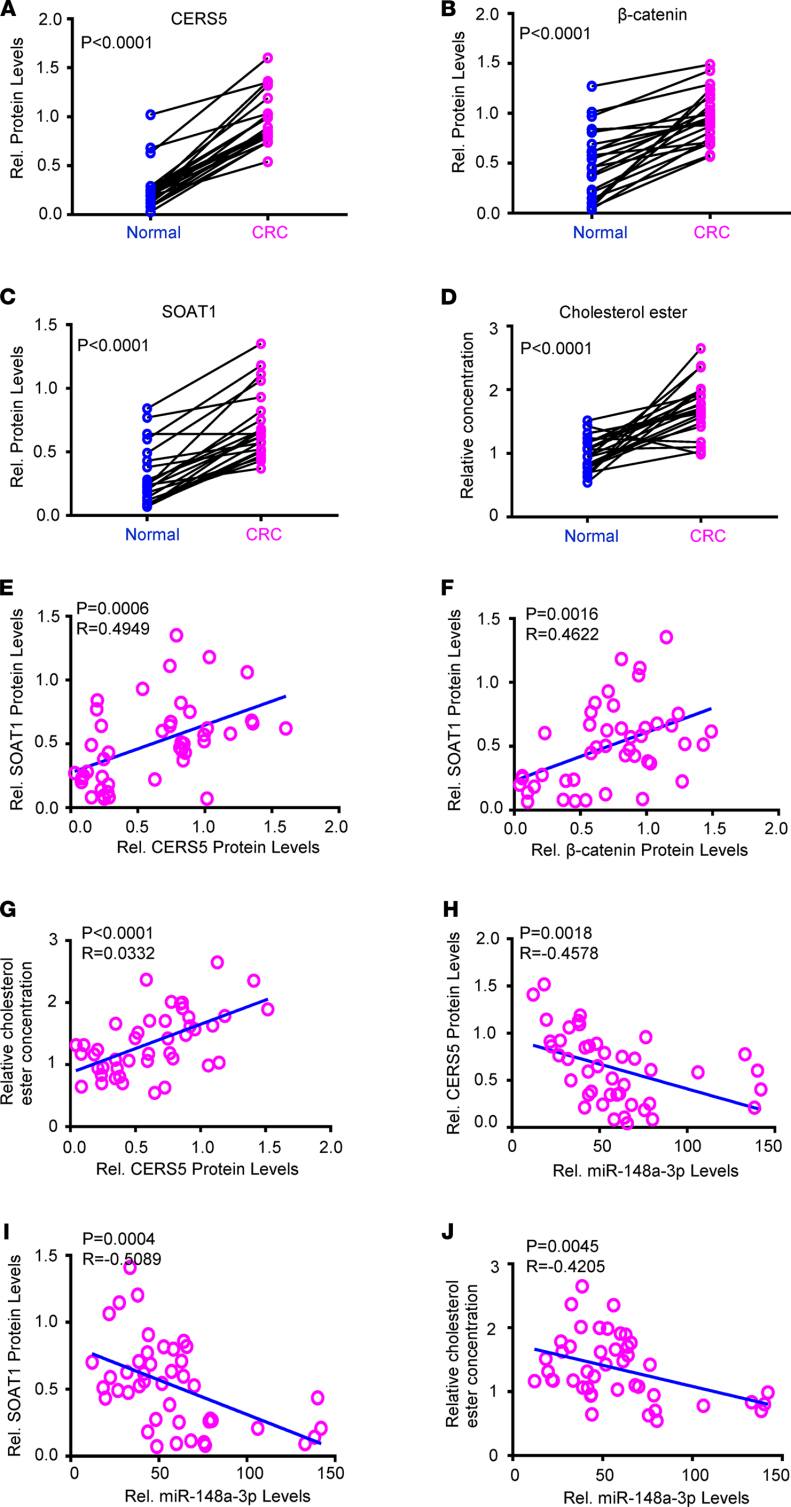
The CERS5/ceramide/β-catenin/SOAT1 signaling axis was dysregulated in human CRC samples. (**A**–**C**) The CERS5 (**A**), β-catenin, (**B**) and SOAT1 (**C**) protein levels in CRC samples compared with normal colon tissues (*n* = 22/group). (**D**) The cholesterol ester in human CRC tissues compared with normal colon tissues (*n* = 22/group). (**E** and **F**) The correlation of SOAT1 protein levels with CERS5 protein (**E**) and β-catenin protein (**F**) levels in human colon tissues (*n* = 44/group). (**G**) Correlation of cholesterol ester levels with CERS5 protein levels in human colon tissues (*n* = 44/group). (**H**–**J**) Correlation of CERS5 protein (**H**), SOAT1 protein, (**I**) and cholesterol ester (**J**) levels with miR-148a-3p levels in CRC samples (*n* = 44/group). (Data were presented as mean ± SEM in **A**–**D**.) (**A**–**D**) Statistical significance was calculated by using 2-tailed matched-pair test. (**E**–**J**) Each circle represents an individual sample.

Next, quantitative immunoblotting was performed on 22 primary human CRCs (Supplemental Figure 7). Results indicated that CERS5, β-catenin, and SOAT1 were all significantly increased in CRCs when compared with their adjacent normal epithelium (Figure 7, A–C). A similar level of increase was also observed in cholesterol ester (Figure 7D).

SOAT1 level was positively correlated with levels of CERS5 and β-catenin (Figure 7, E and F), while CERS5 levels were also positively correlated with that of cholesterol ester in the CRC samples (Figure 7G). However, miR-148a-3p level was negatively correlated with levels of CERS5, SOAT1, and cholesterol ester in CRC samples (Figure 7, H–J). Together, all these results were consistent with the proteomic and biochemical findings shown in our early results above, which supported our conclusion that a CERS5/ceramide/β-catenin/SOAT1 signaling axis existed to mediate cholesterol ester synthesis to play a critical role in CRC of both mice and humans.

### Targeting SOAT1 significantly suppresses both spontaneous and chemical-induced colorectal carcinogenesis.

**Figure 8:**
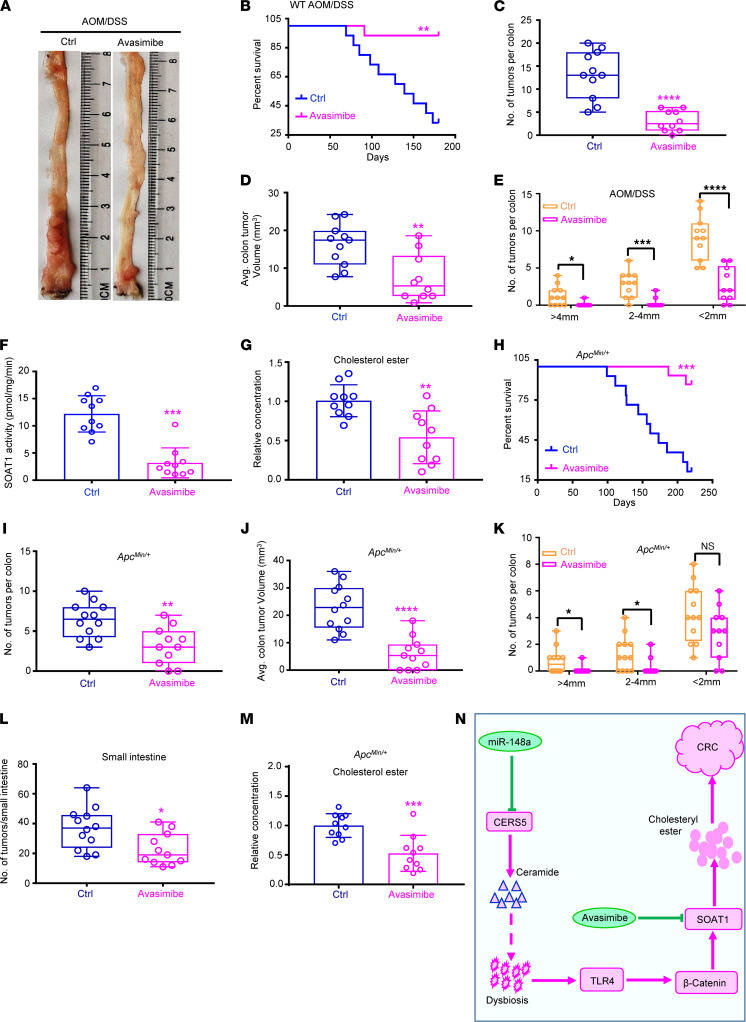
Targeting SOAT1 significantly suppresses spontaneous and chemical-induced colorectal carcinogenesis. (**A**) Representative images of colon tumors subjected to the treatment with control or avasimibe after AOM/DSS induction for 120 days. (**B**) Survival rates of indicated mice subjected to the indicated treatment (*n* = 15/group). (**C**–**E**) Colon tumor numbers (**C**), average colon tumor volumes, (**D**) and tumor sizes (**E**) from **A** (*n* = 10–11/group). (**F** and **G**) The SOAT1 activity (**F**) and relative cholesterol ester level (**G**) from **A** (*n* = 10/group). (**H**) Survival rate of *Apc^Min/+^* mice subjected to the indicated treatment (*n* = 15/group). (**I**–**K**) Colon tumor numbers (**I**), average colon tumor volumes, (**J**) and tumor sizes (**K**) of *Apc^Min/+^* mice from indicated treatment (*n* = 11–12/group). (**L**) The small intestine tumor numbers of *Apc^Min/+^* mice from indicated treatment (*n* = 11–12/group). (**M**) The levels of cholesterol ester in CRCs from **I** (*n* = 10/group). (**N**) Schematic diagram. Depletion of miR-148a induced CERS5-mediated ceramide synthesis. Then, ceramide enhanced β-catenin activity through promoting gut dysbiosis–mediated Tlr4 activity. Next, β-catenin upregulated SOAT1 expression via directly binding to its promoter. Finally, SOAT1 enhanced cholesterol ester synthesis to promote colorectal tumorigenesis, and the SOAT1 inhibitor (avasimibe) had a significant level of therapeutic effect on intestinal cancer. (Data were presented as mean ± SEM in **C**–**G** and **I**–**M**.) *P < 0.05, **P < 0.01, ***P < 0.001, ****P < 0.0001. Statistical significance was calculated by using 2-tailed unpaired *t* test.

Our above results showed that SOAT1 was highly expressed in CRC of both mice and humans and that deletion of SOAT1 could significantly inhibit the growth of both AOM/DSS-induced and spontaneous intestinal cancer in *Apc^Min/+^ mice*. Based on these findings, we next investigated whether the SOAT1 inhibitor (avasimibe) could have a therapeutic effect on AOM/DSS-induced CRC in mice. To determine the therapeutic potential of SOAT1 inhibitor, avasimibe was used to treat AOM/DSS-induced CRCs (Supplemental Figure 8A). Results revealed that lengths of survival were significantly prolonged in the mice with CRC treated with avasimibe and colon tumors were significantly repressed in terms of tumor numbers, volumes, and diameters (Figure 8, A–E), which were consistent with our early results above. Furthermore, avasimibe treatment also significantly inhibited the levels of SOAT1 activity, total cholesterol, and cholesterol ester in AOM/DSS-induced CRCs (Figure 8, F and G, and Supplemental Figure 8B).

To confirm the above results, we treated *Apc^Min/+^* mice with avasimibe and detected the levels of their survival and intestinal tumor numbers (Supplemental Figure 8C). Remarkably, we also found that the averages of total survival lengths were similar for both *Apc^Min/+^* mice and the mice with AOM/DSS-induced CRC because avasimibe reduced the tumor growths in both colon and small intestine in the *Apc^Min/+^* mice (Figure 8, H–L, and Supplemental Figure 8D). Avasimibe treatment also significantly inhibited the levels of SOAT1 activity, total cholesterol, and cholesterol ester levels in the *Apc^Min/+^* mice (Figure 8M and Supplemental Figure 8, E and F). Collectively, these data demonstrated that treatment with avasimibe as a SOAT1 inhibitor could effectively repress both spontaneous and chemical-induced colorectal carcinogenesis and prolong survival in mice.

## Discussion

In this study, we have investigated the role of miR-148a in regulating microbial communities through CERS5-mediated ceramide synthesis (Figure 8N). Our study contributes potentially novel information to realize the results from interaction between miR and ceramide as a mechanism for inducing alterations in the gut microbiota during colorectal tumorigenesis. Despite miR-148a being known as a tumor suppressor in CRC, its function and the underlying molecular mechanisms of miR-148a in gut microbiota dysregulation–mediated CRC have not been uncovered yet (22). Collectively, there were more colorectal tumors in the separately housed *miR-148a^–/–^* mice than in the littermate WT mice. However, there were similar numbers of colorectal tumors in both cohoused *miR-148a^–/–^* mice and their littermate WT mice. These results suggested that the expanded bacteria in *miR-148a^–/–^* mice could play a critical role during colorectal cancer development. Our results can be supported by some recent studies that reported miR-148a regulated lipid metabolism, glucose metabolism, and cholesterol metabolism to control tumor growth (21, 47, 48). Previously, mass spectrometry data also indicated that various types of ceramide were significantly higher in sera of patients with CRC compared with those in control groups (49). It was also shown that ceramides were significantly upregulated in the CRC tissues, associating with CRC patient staging and prognosis (31, 49). Some other studies also suggested that the ceramide could inhibit the growth of CRC through inducing apoptosis of CRC cells (50). However, these previous studies mainly investigated the in vitro phenomenon instead of the in vivo one. Since the occurrence of tumors can be affected by many factors, tumor cells cultured in vitro cannot completely represent the real environment for tumor growth in vivo. Remarkably, our present results suggest that C16:0 ceramide induces alterations in the gut microbiota to promote colorectal tumorigenesis, rather than directly contributing to tumor growth. Particularly, our results suggest that ceramide can regulate the growth of intestinal cancer in mice through altering gut microbiota.

Many recent studies showed that the elevation of serum ceramides could be significantly associated with gut dysbiosis–induced metabolism disorders, inflammation, and diabetes (51–53). In addition, ceramide was also found to regulate skin microbiota balance and growth of *Staphylococcus aureus* (a species of Firmicutes) that affects both healthy and diseased skin (54, 55). In this study, we further revealed that C16:0 ceramide could substantially increase gut dysbiosis to promote colorectal tumorigenesis in mice (Figure 8N). However, the specific molecular mechanism underlying the ceramide-affected alterations in the gut microbiota has not been well understood. Our preliminary results indicated that ceramide could alter the expression of AMPs in intestinal cells, which suggested to us to further pursue how ceramide acts in some related processes of tumorigenesis. In addition, we also found through a combination of genomic, transcriptomic, biological, clinical sample, and mouse model studies that the ceramide/β-catenin/SOAT1 signaling pathway was activated in both CRC patients and mice (Figure 8N). Overall, this finding further supported that miR-148a deficiency can upregulate the ceramide-induced alterations in the microbiota, which next activates the β-catenin/SOAT1 signaling pathway to play its key role during colorectal tumorigenesis. Our finding thus provides the information helpful to study the potential target for therapy for colon cancer.

It was reported that the occurrence of CRC was mainly caused by hyperactivation of the β-catenin signaling pathway (56, 57). However, directly repressing β-catenin was found to have on-target toxicity to humans, and there is still no candidate for a drug target to treat CRC (56, 58). Therefore, the treatment of CRC caused by hyperactivation of β-catenin can only be achieved by targeting its key downstream targets. A recent study suggested that β-catenin regulates cholesterol de novo synthesis through activating the mevalonate pathway in cancer (59). Meanwhile, cholesterol and cholesterol ester also regulated Wnt/β-catenin signaling activity through differential mechanisms (60, 61). However, it is still unknown whether β-catenin mediates cholesterol esterification, contributing to CRC growth. Thus, our current finding successfully demonstrated that β-catenin regulated cholesterol esterification via directly binding to SOAT1 promoter and upregulating its expression in CRC (Figure 8N).

Previously, the SOAT1 inhibitor avasimibe was reported to have reliable therapeutic effect through reducing the incidence of cancers, including liver cancer, pancreatic cancer, glioma, ovarian cancer, and lung carcinoma (41, 62–65). Meanwhile, combined therapy of SOAT1 inhibitor avasimibe plus programmed cell death 1 antibody was found to have an enhanced therapeutic effect in controlling some cancer growth in mice (66). However, it is still unclear whether avasimibe has any therapeutic effect on intestinal cancer. Here, our current data support that avasimibe can be used as the effective treatment for both AOM/DSS-induced cancer and spontaneous intestinal cancer of *Apc^Min/+^* mice. Therefore, our data supply encouraging information for potential clinical application to use SOAT1 inhibitor (avasimibe) to treat those CRC patients with high activity of β-catenin pathway.

## Methods

### Sources of stable cell lines and cell culture.

All stable cell lines were obtained from the American Type Culture Collection, including human embryonic kidney HEK293 cells immortalized with human telomerase reverse transcriptase and human colon tumor–derived cell lines HCT116 and SW480. All cells were regularly authenticated by short tandem repeat analysis and tested for absence of *Mycoplasma* contamination. They were used within 5 passages after thawing. Cells were cultured as previously described (22).

Stable cell lines expressing the indicated vectors were generated by retroviral or lentiviral transduction in the presence of 8 μg/mL polybrene followed by selection with blasticidin, puromycin, or G418 (all from Thermo Fisher Scientific) for at least 10 days. After modifications, stable cell lines were examined for the expression of the indicated vectors by real-time PCR or Western blot.

### Human CRC samples.

CRC samples were obtained and used as previously described (22). Written informed consent was given by all patients at the Union Hospital in Wuhan, China. The studies were conducted in accordance with Declaration of Helsinki and approved by the review board of Wuhan University. The diagnoses of all samples were confirmed by professional histological experts.

### Plasmids and constructs.

The open reading frame of human β-catenin was amplified and subcloned into pCMV-HA vectors. Human β-catenin and mouse *Cers5* shRNA vectors were subcloned into PLKO.1 vector. Adenovirus vector was supplied by Yan Wang (College of Life Sciences, Wuhan University). Primers and shRNAs used in this study are listed in Supplemental Tables 1 and 2.

### Immunoblot.

Transfected cells and CRC tissues were lysed in lysis buffer (20 mM Tris at pH 7.4, 300 mM NaCl, 1% Triton X-100, 1 mM EDTA, 10 mg/mL aprotinin, 10 mg/mL leupeptin, and 1 mM PMSF). Immunoblot assay was performed as previously described (66). Antibodies used in this study were listed in Supplemental Table 3.

### Reporter, ChIP, and qPCR assays.

Human SOAT1 promoter sequences were inserted into pGL3-basic luciferase vector (Promega). Luciferase assays were performed as described previously (22).

We found the putative transcription factor binding sites within the SOAT1 and USP19 promoter regions (–5 to +5 kb) in the MotifMap website. The URL of the MotifMap website is http://motifmap.ics.uci.edu/ ChIP was performed as described previously (22). In brief, intracellular protein-DNA complexes were cross-linked with 1% formaldehyde, sonicated, and subjected to ChIP using specific antibodies. After reversal of cross-links, precipitated DNA was purified and analyzed by qPCR with the primers indicated in Supplemental Table 4.

For RT-qPCR, total RNA was isolated using TRIzol (Invitrogen) followed by DNase (Thermo Fisher Scientific) treatment. Reverse transcription was performed with a cDNA Synthesis Kit (Promega), and qPCR was performed using SYBR Green Master Mix (Bio-Rad) using standard protocols. All primer sequences used are shown in Supplemental Table 5. β-Actin was used as an internal control.

### Luciferase assays.

Luciferase assays were performed as described previously (22). The targets of miR-148a 3′UTRs and Pri-miR-148a promoter regions were amplified from human genomic DNA, then inserted into pGL3-basic vector with the HSV-TK promoter. Mutations were generated by overlap extension PCR. HCT116 and RKO CRC cells were transfected with the indicated firefly luciferase reporter plasmid, Renilla reporter plasmid as a normalization control, and miR-148a or a control vector, miR-148a-3p sponge, or control miR sponge. Then the luciferase activity was measured and analyzed using the Dual-Luciferase Reporter Assay System (Promega).

### Metabolic assays.

For the cholesterol ester assay, 10^6^ cells or 2 mg tissues were harvested, and cholesterol ester concentrations were detected by using Cholesterol Ester Quantification kit (ab65359, Abcam) according to the manufacturer’s instructions.

For the total cholesterol assay, 10^6^ cells or 2 mg tissues were harvested, and total cholesterol concentrations were detected by using total Cholesterol Quantification kit (JW.MO2308, GIVEI) according to the manufacturer’s instructions.

### 16s rDNA genomic DNA qPCR analysis.

This was performed as described previously (8, 20). Bacterial genomic DNA was isolated from frozen fecal samples by using stool DNA Extraction Kit (PHYGENE). To detect the relative level of gut bacteria, 16s rDNA was amplified from bacterial genomic DNA using iTaq Universal SYBR Green Supermix (Bio-Rad). qPCR primer sequences of 16s rDNA are listed in Supplemental Table 6.

### Animal experiments.

WT and *Apc^Min/+^* mice were generated from species of C57BL/6 mice from Model Animal Research Center of Nanjing University. The *Soat1^fl/fl^* mice were generated from C57BL/6 mice from Baoliang Song’s laboratory (Wuhan University, Wuhan, China) and Boliang Li’s laboratory (Shanghai Institute of Biochemistry and Cell Biology, Chinese Academy of Sciences, Shanghai, China). The *Tlr4^–/–^* mice were also generated from C57BL/6 mice from Hongliang Li’s laboratory (Wuhan University, Wuhan, China). All animal studies were approved by the Animal Care Committee of Wuhan University. AOM/DSS-induced CRCs were generated following the previously described procedures (22). Briefly, 8-week-old female C57BL/6 mice were injected intraperitoneally with 10 mg/kg AOM (MilliporeSigma). Seven days later, mice were given drinking water containing 2.5% DSS (MP Biomedicals) for 7 days followed by 2 weeks of regular drinking water. Afterward, mice were fed with 3 rounds of 2.5% DSS water for 1 week and sacrificed on day 120.

For administration of adenovirus to AOM/DSS-induced CRC mice, C57BL/6 mice were randomly distributed into different groups of about 15 animals in each. Concentrated viruses in a volume of 100 μL were delivered intraperitoneally twice per week for 3 weeks. At day 120, tumor burden was evaluated after sacrificing the mice.

Antibiotics treatment of mice was performed by supplementation of drinking water with ampicillin (1 mg/mL), gentamicin (1 mg/mL), metronidazole (1 mg/mL), neomycin (1 mg/mL), and vancomycin (0.5 mg/mL).

Investigators who measured the mice were blinded to the treatment groups. Mice were grouped into control group and SOAT1 inhibitor (avasimibe) treatment group. Both control and avasimibe treatment groups were given intraperitoneal injection every 3 days with a dosage of 15 mg/kg for 3 months (67).

### Bacterial culture from CRC tissues and gut microbiota mix.

The stocks of cecal contents from mice were serially diluted with PBS and seeded onto nonselective agar plates (blood liver agar plates from Eiken Chemical or Eggerth-Gagnon [EG] agar plates). EG agar plates contained the following components (quantities expressed per liter): Lab-Lemco Powder (2.8 g, Oxoid), proteose peptone no. 3 (10.0 g, Difco), yeast extract (5.0 g, Difco), Na_2_HPO_4_ (4.0 g), d1-glucose (1.5 g), soluble starch (0.5 g), l-cystine (0.2 g), l-cysteine-HCl-H_2_O (0.5 g), Tween 80 (0.5 g), Bacto agar (16.0 g, Difco), and defibrinated horse blood (50 mL). After culture under aerobic or anaerobic conditions at 37°C for 2 or 4 days, all colonies were picked up to be placed into the same tube, then cultured for an additional 2 or 4 days at 37°C in ABCM broth (Eiken Chemical). Then all strains were collected as gut microbiota mix.

### AGO2 RNA immunoprecipitation.

RNA immunoprecipitation (RIP) was done using the protocol described previously (22). Briefly, tissues derived from WT and *miR-148a^–/–^* colon tissues were UV irradiated and lysed with RIP buffer containing RNase Inhibitor (EO0381, Thermo Fisher Scientific) and proteinase inhibitor (MilliporeSigma) and then treated with DNase I (Thermo Fisher Scientific). The separated supernatant was incubated with 1 mg rabbit mono-antibody against AGO2 (C34C6, Cell Signaling Technology) or control IgG for 4 hours and then added to protein G beads (Life Technologies). After digestion of protein, the precipitated RNA was purified using TRIzol reagent (Life Technologies) and analyzed by RT-qPCR.

### Lipidomics assays.

CRC sections were quickly dissected and snap-frozen in liquid nitrogen with a precooled Wollenberger clamp. Frozen samples were ground in liquid nitrogen and then placed into 1 mL of 0.3 M KOH in 90% methanol at 80°C for 1 hour in a 2 mL glass vial for saponification. Formic acid (0.1 mL) was added for neutralization. The saponified fatty acids were extracted with 0.5 mL of hexane and vortexed, and the top organic layer was transferred to a new glass vial. Samples were then dried under a stream of N_2_ and dissolved in 1 mL of isopropanol/methanol (1:1, v/v) solution for liquid chromatography–tandem mass spectrometry (LC-MS) analysis. Separation was performed by reversed-phase ion-pairing chromatography on a C8 column coupled to negative-ion mode, full-scan LC-MS at 1 Hz scan time and 100,000 resolving power (stand-alone orbitrap; Thermo Fischer Scientific) (68).

### SOAT1 enzyme activity assay.

SOAT1 activity was measured in microsomes isolated from colon samples taken at the beginning and end of perfusion. Colon biopsies were taken from a different region of each colon, although we found no regional differences in SOAT1 activity in control colon. Colon samples (200–500 mg) were homogenized in 3 mL ice-cold buffer containing 0.1 M K_2_HPO_4_, 0.25 M sucrose, and 1 mM EDTA at pH 7.4 using a Polytron homogenizer (Brinkmann Instruments) set at an intermediate speed for 10–15 seconds. The homogenate was centrifuged for 15 minutes at 12,000*g* (4°C) to remove cell debris, and the supernatant was then recentrifuged for 60 minutes at 100,000*g* (4°C). The resulted microsomal pellet was suspended in a small volume (<1 mL) of assay buffer containing 0.1 M K_2_HPO_4_ at pH 7.4 to yield a protein concentration of 1–4 mg/mL. The microsomal preparation was immediately frozen in liquid nitrogen and stored for up to 2 weeks at –80°C. Freezing the microsomes was shown not to affect SOAT1 activity (39, 69). Optimal assay conditions were determined by using [1-^3^H] oleate and excess free cholesterol as substrates. The addition of 50 nmol exogenous cholesterol per assay in detergent was intended to eliminate the effect of substrate availability on enzyme activity. The nonionic detergent Tyloxapol (Sigma Chemical Co.) was used to solubilize the cholesterol, with a final detergent concentration in the assay of 0.25%. The standard SOAT1 assay started with a 30-minute preincubation at 37°C containing 200 μg microsomal protein, 1.0 mg bovine serum albumin, and 50 nmol exogenous cholesterol. The reaction was initiated by the addition of 30 nmol [1-^3^H] oleate (5000–10,000 dpm/nmol) in a final volume of 300 μL. After a 2-minute incubation period, the reaction was stopped by the addition of 6 mL chloroform/methanol (2:1, v/v) containing 15 μg cholesteryl oleate as a carrier. The phases were separated by the addition of 1.2 mL of 0.88% KCl, and an aliquot of the lower phase was removed. Cholesteryl esters were then isolated by thin layer chromatography for determination of total ^3^H radioactivity by scintillation spectrometry (39, 69, 70).

### Bioinformatic analysis.

CRC data sets were downloaded from TCGA data portal (http://tcga-data.nci.nih.gov). Gene expression was assessed and Kaplan-Meier curves were analyzed in human CRC tissues from the TCGA RNA-Seq data set (*n* = 378). CRC samples were assigned to 2 groups based on gene expression level using the minimum *P* value approach.

### Statistics.

Experimental data were analyzed using the Wilcoxon signed rank test, 1-way ANOVA, or 2-tailed Student’s unpaired *t* test, and the correlation was analyzed using a Spearman rank correlation test. Kaplan-Meier curves for survival were analyzed with GraphPad software using the log-rank test. Results are represented as the mean ± SEM or mean ± SD. In figures, the box plots depict the minimum and maximum values (whiskers), the upper and lower quartiles, and the median. The length of the box represents the interquartile range. *P* < 0.05 was considered statistically significant.

### Study approval.

All animal studies were approved by the Animal Care Committee of Wuhan University and were conducted according to the Wuhan University Regulations for Animal Experiments.

## Author contributions

YZ and YL designed the study; YZ and LG performed most of the experiments; Xi Lin constructed plasmids; CL and BL performed some animal experiments; and YZ, LG, Xi Lin, JZ, YT, XZ, BL, Xingrong Lin, CL, EBP, and YL discussed the results. YZ and YL wrote the manuscript with comments from all authors.

## Supplementary Material

Supplemental data

## Version 1 12/16/2021

In-Press Preview

## Version 2 02/08/2022

Electronic publication
