# Supplementary material for: Ceramide-mediated gut dysbiosis enhances cholesterol esterification and promotes colorectal tumorigenesis in mice
Source: JCI Insight. 2022 Feb 8;7(3):e150607. doi: 10.1172/jci.insight.150607 (PMC8855812; doi:10.1172/jci.insight.150607)
Supplement: Supplemental data [file jciinsight-7-150607-s016.pdf]

Supplementary Materials

Ceramide-mediated Gut Dysbiosis Enhances Cholesterol Esterification and Promotes Colorectal Tumorigenesis in Mice

Yahui Zhu, Li Gu, Xi Lin, Jinmiao Zhang, Yi Tang, Xinyi Zhou, Bingjun Lu, Xingrong Lin, Cheng Liu, Edward V. Prochownik, Youjun Li

Supplemental figures

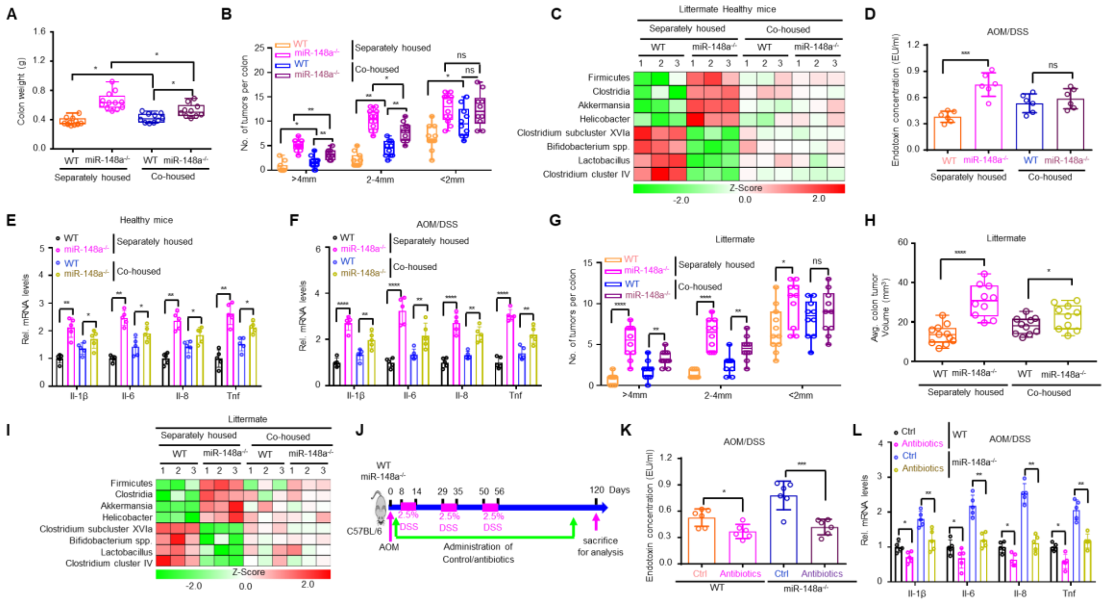

Supplemental Figure 1. Depletion of miR-148a increased gut dysbiosis to enhance colorectal tumorigenesis. (**A and B**) The colon weights (A) and tumor diameters (B) of both WT and *miR-148a*$^{-/-}$ mice in either separately housed or co-housed from Figure 1A (n = 10–12/group). **(C)** qPCR assays on the relative levels of indicated gut microbiota of either separately housed or co-housed littermates of both WT and *miR-148a*$^{-/-}$ mice before any treatment (n = 3/group). **(D)** The endotoxin concentrations in either separately housed or co-housed WT and *miR-148a*$^{-/-}$ mice from Figure 1A (n = 6/group). **(E and F)** The transcript levels of *Il-1*β, *Il6*, *Il8* and *Tnf* were determined by reverse transcription quantitative-PCR in colon tissues collected from separately housed or co-housed WT and *miR-148a*$^{-/-}$ mice before (E) and after (F) AOM/DSS treatment (n = 5/group). **(G and H)** Tumor diameters (G) and average volumes (H) of Colon tumor in mice from indicated treatment (n = 10–11/group). **(I)** qPCR assays on the relative levels of indicated gut microbiota of mice from Supplemental Figure 1G (n = 3/group). (**J**) Schematic overview of treatment regimen of AOM/DSS-induced CRCs. **(K)** The endotoxin concentrations in WT and *miR-148a*$^{-/-}$ mice from Figure 1D (n = 6/group). **(L)** The transcript levels of *Il-1*β, *Il6*, *Il8* and *Tnf* were determined by reverse transcription quantitative-PCR in CRC tissues collected from Figure 1D (n = 5/group). Data were

presented in mean ± SEM for A, B, D, E, F, G, H, K and L. *p<0.05, **p<0.01, ***p<0.001, ****p<0.0001. Statistical significance was calculated by using one-way ANOVA (**A** and **B**) or two-tailed unpaired t test (**D**, **E**, **F**, **G**, **H**, **K** and **L**).

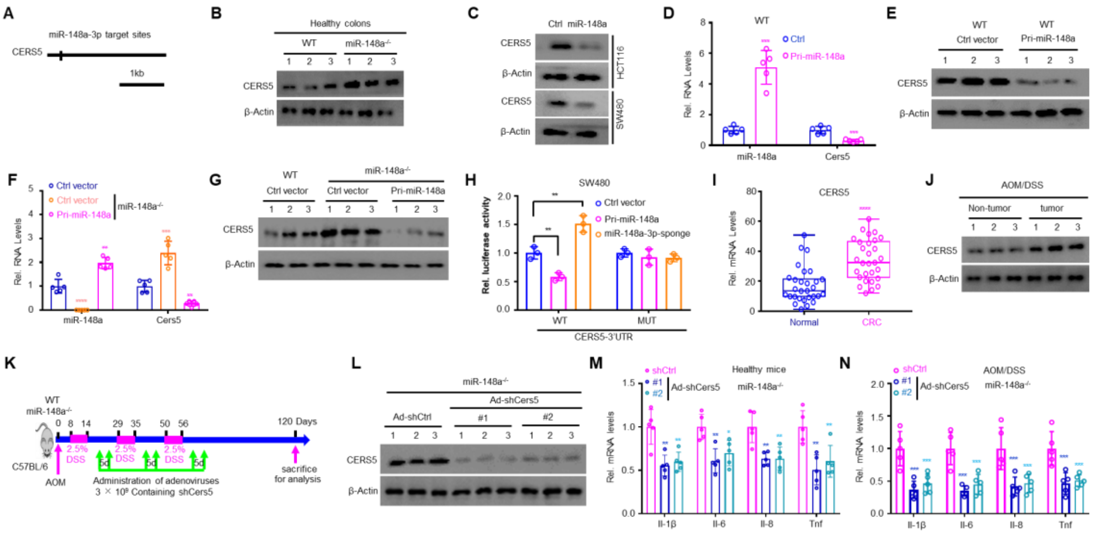

Supplemental Figure 2. Depletion of miR-148a upregulated CERS5 expression to increase colorectal tumorigenesis. (A) Schematic of human *CERS5* 3′ UTRs. The predicted miR-148a-3p binding sites. **(B)** Western blot was used to detect the CERS5 protein levels in colon of WT and *miR-148a*$^{-/-}$ before any treatment (n = 3/group). **(C)** The protein levels of CERS5 in CRC cells transfected with control or miR-148a. (**D and E**) The mRNA (D) (n = 5/group) and protein (E) (n = 3/group) levels of CERS5 in colon tissues collected from WT mice administrated with indicated lentivirus. (**F and G**) The mRNA (F) (n = 5/group) and protein (G) (n = 3/group) levels of CERS5 in the colon tissues collected from WT and *miR-148a*$^{-/-}$ mice administrated with indicated lentivirus. (**H**) Luciferase activity of the reporter vector containing the WT or miR-148a-3p binding mutant 3′ UTR of CERS5 was determined after cotransfection with indicated vector in SW480 cells. (**I**) The CERS5 expression in 29 pairs of CRCs from Wuhan Union Hospital (n = 29/group). **(J)** The protein levels of CERS5 in non-tumor and tumor tissues after AOM/DSS induction (n = 3/group). **(K)** Schedule of adenoviruses treatment during AOM/DSS-induced CRC tumorigenesis in mice. (**L**) Western blot was used to detect the CERS5 protein expression in AOM/DSS-induced mouse CRC tissues treated with the indicated vector (n = 3/group). **(M and N)** The transcript levels of *Il-1β*, *Il6*, *Il8* and *Tnf* were determined by quantitative-PCR in colon tissues collected from *miR-148a*$^{-/-}$ mice treated with indicated adenovirus before (M) and after (N) AOM/DSS induction (n = 5/group). Data in D, F, H, I, M and N were presented in mean ± SEM. *p<0.05, **p<0.01, ***p<0.001, ****p<0.001. Statistical significance was calculated by using two-tailed

unpaired t test. Data shown in H are representatives of 3 independent experiments.

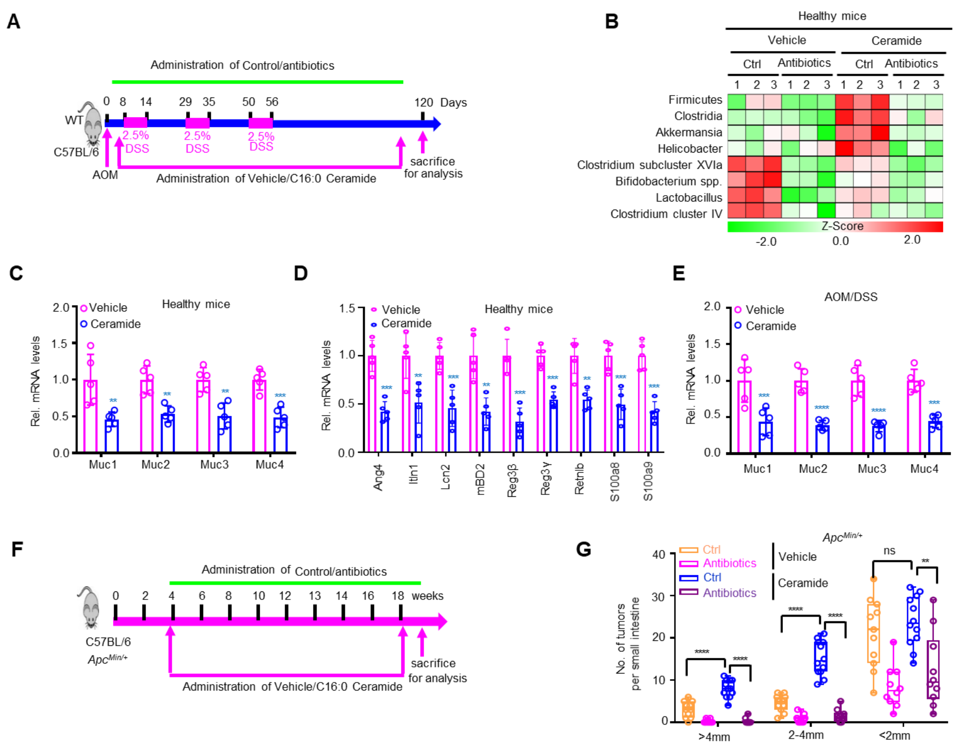

Supplemental Figure 3. Ceramide increased gut dysbiosis to promote colorectal tumorigenesis. (**A**) The experimental scheme showed how AOM/DSS induced colorectal cancer with the indicated treatments. **(B)** qPCR analyses on Bacterial 16S rRNA indicated gut microbiota from the native mice with indicate treatment (n = 3/group). **(C and D)** The relative mRNA levels of mucus (C) and AMPs (D) of vehicle or ceramide treated healthy mice by qPCR analysis (n = 5/group). **(E)** The relative mRNA levels of mucus of mice from indicated treatment by qPCR analysis (n = 5/group). (**F**) The experimental scheme showed how *Apc*$^{Min/+}$ mice developed CRC with the indicated treatments. (**G**) The small intestine tumor diameters of mice from Supplemental Figure 3F (n = 10–12/group). Data were presented as mean ± SEM in C, D, E and G. **p<0.01, ***p<0.001, ****p<0.001. Statistical significance was calculated by using one-way ANOVA (**G**) or two-tailed unpaired t test (**C**, **D**, **E**).

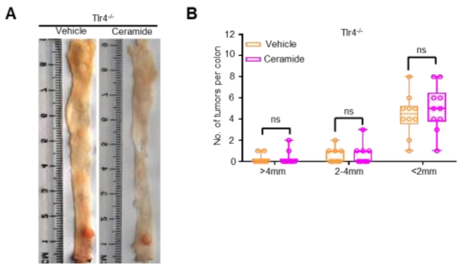

Supplemental Figure 4. miR-148a depletion promoted CRC growth dependent on ceramide-mediated alterations in the gut microbiota to activate TLR4.

(A) Representative images of colon tumors from *Tlr4*$^{-/-}$ + vehicle, *Tlr4*$^{-/-}$ + ceramide mice in AOM/DSS-induced CRC. (**B**) Colon tumor sizes from Supplemental Figure 4A (n = 10/group). Data were presented in mean ± SEM in B. Statistical significance was calculated by using two-tailed unpaired t test.

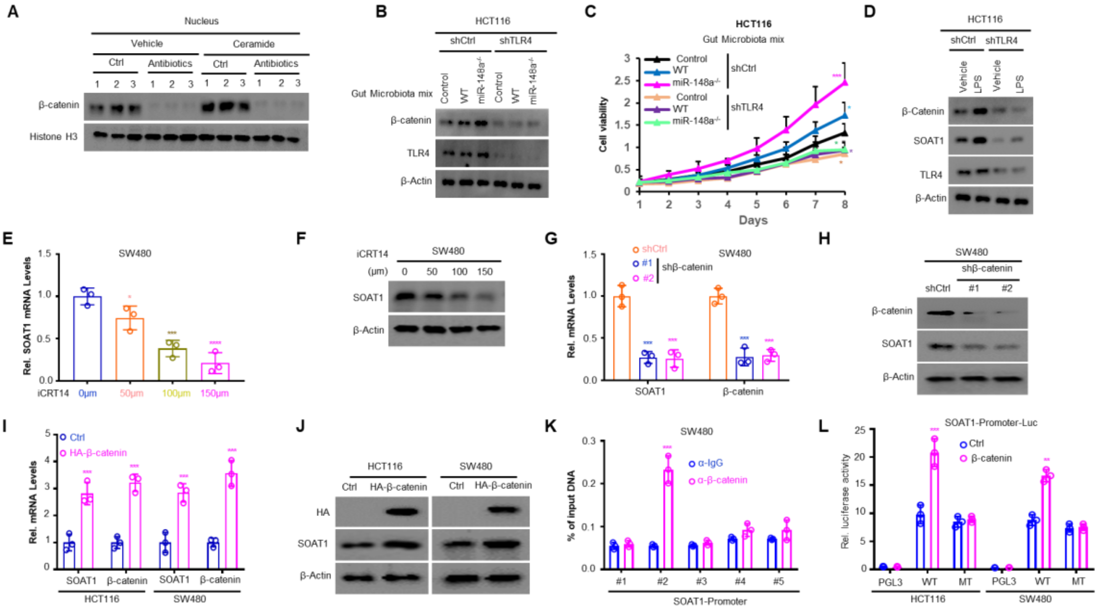

Supplemental Figure 5. SOAT1 was transcriptionally activated by β-catenin/TCF1 complex. (A) Western blot was used to detect β-catenin expression in the nuclear extract of CRCs from vehicle or ceramide treated mice (n = 3/group). **(B)** The protein levels of β-catenin in HCT116 cells after transfection with shCtrl or sh*TLR4* were treated with the gut microbiota from WT or *miR-148a*$^{-/-}$ mice. **(C)** MTT assay was used to detect on cell viability of HCT116 cells from Supplemental Figure 5B. **(D)** The protein levels of β-catenin and SOAT1 in HCT116 cells with indicated treatments. (**E and F**) The mRNA (E) and protein (F) levels of SOAT1 in SW480 cells were treated with β-catenin inhibitor (iCRT14). (**G and H**) Depletion of β-catenin decreased the mRNA (G) and protein (H) levels of SOAT1 in SW480 cells. (**I and J**) mRNA (I) and protein levels (J) of SOAT1 in HCT116 and SW480 cells after β-catenin overexpression. (**K**) ChIP-qPCR analysis of the *SOAT1* promoter in SW480 cells. (**L**) Luciferase activity of *SOAT1* promoter in HCT116 and SW480 cells after β-catenin overexpression. Data were presented as mean ± SD in C. Data were presented as mean ± SEM in E, G, I, K and L. *p<0.05, **p<0.01, ***p<0.001, ****p<0.001. Statistical significance was calculated by using two-tailed unpaired t test. Data shown in B-L are representatives of 3 independent experiments.

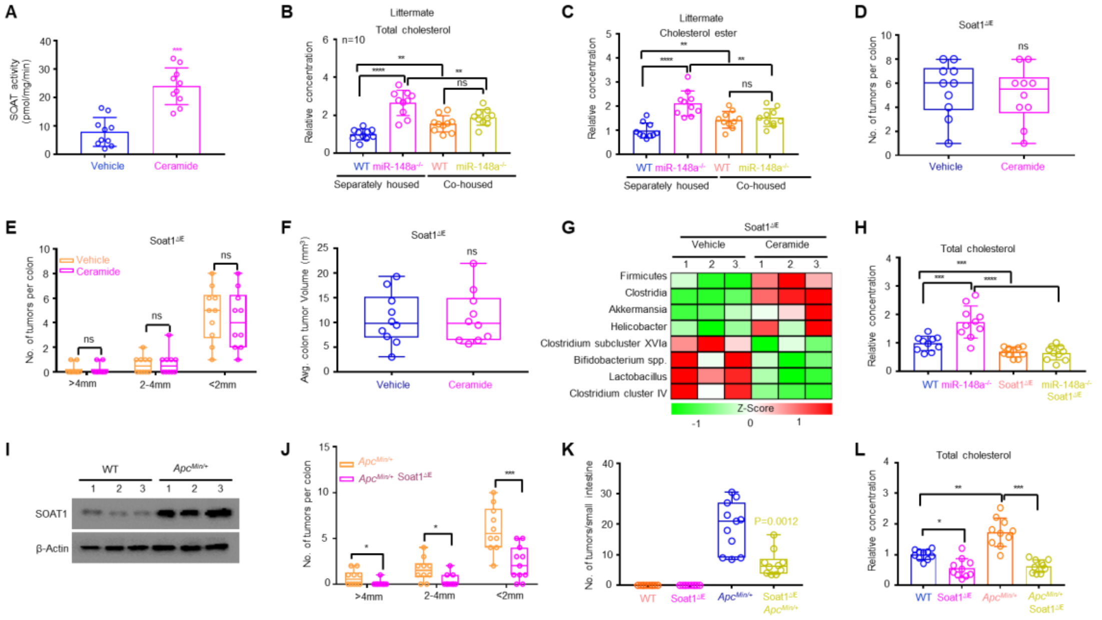

Supplemental Figure 6. *Soat1* loss attenuated intestine tumorigenesis in *miR-148a*$^{-/-}$ and *Apc*$^{Min/+}$ mice. (**A**) The SOAT1 activity in CRCs treated with vehicle or ceramide. **(B and C)** The colon total cholesterol (B) and cholesterol ester (C) levels from indicated mice (n = 10/group). (**D-F**) Levels of colon tumor numbers (D), tumor sizes (E) and tumor volumes (F) from *Soat1*$^{\Delta IE}$ + vehicle and *Soat1*$^{\Delta IE}$ + ceramide treated mice in AOM/DSS-induced CRC (n = 10/group). (**G**) qPCR assays on Bacterial 16S rRNA indicated gut microbiota from Supplemental Figure 6D (n = 3/group). **(H)** The colon total cholesterol levels from indicated mice (n = 10/group). (**I**) Western blot was used to detect the SOAT1 protein level in WT and *Apc*$^{Min/+}$ mice (n = 3/group). (**J**) The sizes of colon tumors from *Apc*$^{Min/+}$ and *Soat1*$^{\Delta IE}$*Apc*$^{Min/+}$ mice (n = 10–11/group). (**K**) Small intestine tumor numbers from WT, *Soat1*$^{\Delta IE}$, *Apc*$^{Min/+}$ and *Soat1*$^{\Delta IE}$*Apc*$^{Min/+}$ mice (n = 10–11/group). (**L**) Total cholesterol levels in CRCs from indicated mice (n = 10/group). Data were presented as mean ± SEM in A, B, C, D, E, F, H, J, K and L. *p<0.05, **p<0.01, ***p<0.001, ****p<0.0001. Statistical significance was calculated by using one-way ANOVA (**B**, **C**, **H** and **L**) or two-tailed unpaired t test (**A**, **D**, **E**, **F**, **J** and **K**).

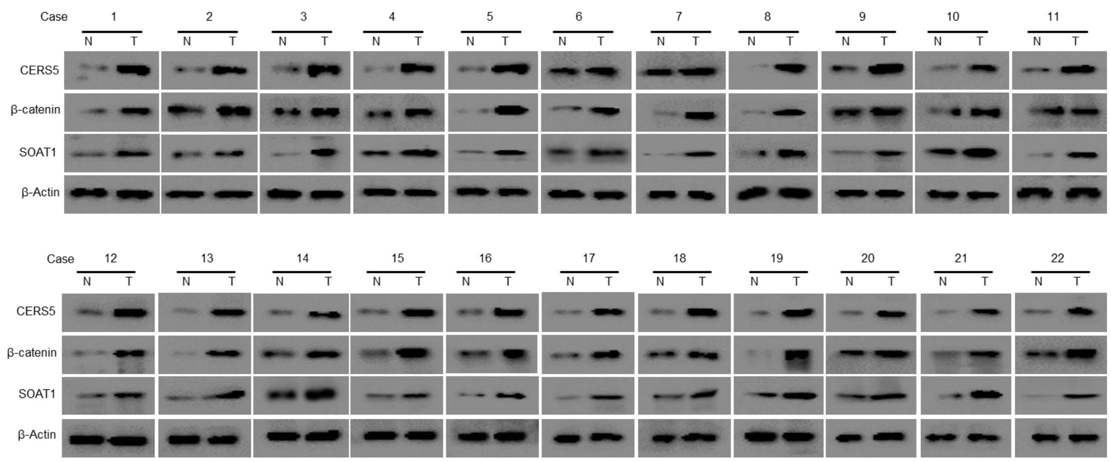

Supplemental Figure 7. The CERS5-Ceramide-β-catenin-SOAT1 signaling axis was dysregulated in human CRC tissues. Western blot assay was used to analyze 22 colorectal cancer patient samples (T) with the adjacent normal colon tissues (N).

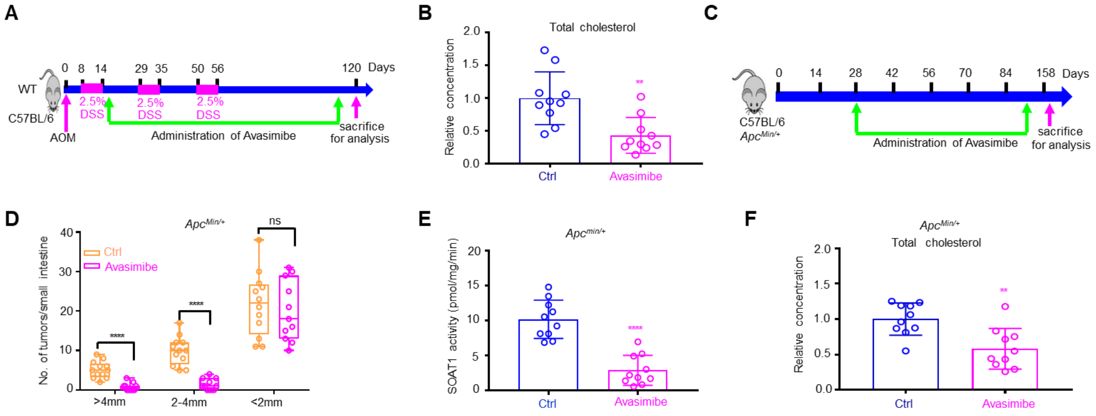

Supplemental Figure 8. Targeting SOAT1 significantly suppressed both spontaneous colorectal carcinogenesis and chemical-induced one. (**A**) Schematic overview of treatment regimen of AOM/DSS-induced WT mice. (**B**) The total cholesterol levels in CRCs from WT mice with indicated treatments (n = 10/group). (**C**) Schematic overview of treatment regimen of *Apc*$^{Min/+}$ mice. (**D**) The small intestine tumor diameters of *Apc*$^{Min/+}$ mice from Supplemental Figure 8C (n = 11–12/group). (**E and F**) The levels of SOAT1 activity (E) and total cholesterol (F) in CRCs from Figure 8C (n = 10/group). Data were presented as mean ± SEM in B, D, E and F. **p<0.01, ****p<0.001. Statistical significance was calculated by using two-tailed unpaired t test.

Supplemental Table 1. ShRNA sequences used in this study.

**shRNA**	**Target Sequences（5’>3’）**
sh*Cers5* #1	GCATGTGGAGATTCACTTATT
sh*Cers5* #2	GATGCTGTTTGAGCGATTTAT
sh*TLR4* #1	CCAAGTAGTCTAGCTTTCTTA
sh*TLR4* #2	CGTTTGGTTCTGGGAGAATTT
sh*β-catenin* #1	TTGTTATCAGAGGACTAAATA
sh*β-catenin* #2	TCTAACCTCACTTGCAATAAT

Supplemental Table 2. **Primer sequence for constructs used in this study.**

Insert	Forward Primer (5’>3’)	Reverse Primer (5’>3’)
CERS5 3’UTR	CGC TCTAGA CCCATATCT ACTCTTCTGT GAT	CGC GGATCC CCATCCATGGTTCTGTTTCTAT
CERS5 3’UTR MUT	CGCTCTAGAGTGATTGGGAGACTGCAAGTAGAGTGAGAGT ATCAAAGAA	CGC GGATCC CCATCCATGGTTCTGTTTCTAT
HA-β-catenin	CGC GTCGAC T GCTACTCAA GCTGATTTGA T	CGCGCGGCCGC TTACAGGTCAGTATCAAACCA
PGL3-*SOAT1*-Promoter	CGC AGATCT CCAATGACAGCCAGTACTAA	CGC AAGCTT CAGGAGATTCCTTACCGT
SOAT1-Promoter MT	GGATGCTGAGGGCCATTTCTGAGGGTCGTGAACAGTGCA	CCCAGCAGGCTGCACTGTTCACGACCCTCAGAAATGGCCCT

Supplemental Table 3. Antibodies used in this study.

Gene	Company	Catalog No	Source	Dilution
CERS5	Abcam	ab73289	Rabbit polyclonal antibody	1:500
SOAT1	Abcam	ab39327	Rabbit polyclonal antibody	1:500
SOAT1	Santa Cruz	sc-69836	mouse monoclonal antibody	1:1000
β-catenin	Abcam	ab6302	Rabbit polyclonal antibody	1:2000
c-Myc	Santa Cruz	A2814	mouse monoclonal antibody	1:500
HA	OriGene	AF4911	mouse monoclonal antibody	1:2000
Flag	SIGMA	F3165	mouse monoclonal antibody	1:5000
β-Actin	Santa Cruz	H1914	mouse monoclonal antibody	1:2000

Supplemental Table 4. ChIP PCR primers for *SOAT1* promoters.

Amplicons	Forward Primer (5'>3')	Reverse Primer (5'>3')
SOAT1 #1	CTCAACATGGTCTACAAACTT	GTAGGAGGAGTTTTGTCATCA
SOAT1 #2	GCACACGTGGCAATCACAGTT	GGTTTAGCAGAACTGACTA
SOAT1 #3	CCTCAGGTAACGGTGGTGCT	GGGCGCCTGACGGTAACTTT
SOAT1 #4	GGATAAAGACCTACTGAGTGA	CTGAGGCAGGTATAAGAGTT
SOAT1 #5	GCTGTCTACTTAGCTTT	CTAGCAGTGAAGGAATCTCTA

Supplemental Table 5. Quantitative PCR primers used in this study.

**Gene**	**Forward Primer (5'>3')**	**Reverse Primer (5'>3')**
β-Actin	ATCATGAAGTGTGACGTGGACAT	AGGAGCAATGATCTTGATCTTCA
Col4a1	CTGGCACAAAAGGGACGAG	ACGTGGCCGAGAATTTCACC
Itga11	TGCCCCAATGGAAACCAATG	CACTCGTGCGACCAGAGAG
Cers5	CGGGGAAAGGTGTCTAAGGAT	GTTCATGCAGTTGGCACCATT
Ltbp1	CCAGTCCCAAGTCTCTTACCA	CTGGAAGCATCGGCCAAGT
Usp32	GCGGCCTCTCCTATTACATGA	ATAGCTCCCGGATTCACTTGA
Il-1β	GAAATGCCACCTTTTGACAGTG	TGGATGCTCTCATCAGGACAG
Il6	CGGCCTTCCCTACTTCACAA	TGCAAGTGCATCATCGTTGT
Il8	CTGCAACAGAAAGGAAGTGAT	AGCTTCATTGCCGGTGGAAA
Tnf	TGATCGGTCCCCAAAGGGAT	CACTTGGTGGTTTGCTACGA
Muc1	AGTGCCAAGTCAATACCCTGT	CTGGGGTGAACTGTTACTGGA
Muc2	AGGGCTCGGAACTCCAGAAA	CCAGGGAATCGGTAGACATCG
Muc3	CCGAGAGCGGAAGTGTGTG	TGTAACTGTGGTTTTGGTCTTCA
Muc4	ACAGGTGTAACTAGAAGCCTCG	CAGGGGTGCTATGCACTACTG
Ang4	GCCCTTATGGAGAAAACTTC	CCATGACAGTGAACGCTGAA
Itln1	CAGCACTTGGGACATAATCTGT	TCCTTCTCCGTATTTCACTGGG
Lcn2	TGGCCCTGAGTGTCATGTG	CTCTTGTAGCTCATAGATGGTGC
mBD2	GGAGGAAGTGATCCGAAAATCAG	AGCATTTCCCAGGTATCTTGC
Reg3β	CCCTCCGCACGCATTAGTT	CAGGCCAGTTCTGCATCAAA
Reg3γ	ATGCTTCCCCGTATAACCATCA	GGCCATATCTGCATCATACCAG

Retnlb	AAGCCTACACTGTGTTTCCTTTT	GCTTCCTTGATCCTTTGATCCAC
S100a8	AAATCACCATGCCCTCTACAAG	CCCACTTTTATCACCATCGCAA
S100a9	ATACTCTAGGAAGGAAGGACACC	TCCATGATGTCATTTATGAGGGC
Soat1	GAAGGCTCACTCATTTGTCAGA	GTCTCGGTAAATAAGTGTAGGCG
SOAT1	CAAGGCGCTCTCTCTTAGATG	GGTCCAAACAACGGTAGGAAA
β-catenin	CATCTACACAGTTTGATGCTGCT	GCAGTTTTGTCAGTTCAGGGA

Supplemental Table 6. **bacteria 16S rDNA primers used in Quantitative PCR of this study.**

Bacteria	Forward Primer (5'>3')	Reverse Primer (5'>3')
Akkermansia	CAGCACGTGAAGGTGGGGAC	CCTTGCGGTTGGCTTCAGAT
Anaerostipes	AAGTCGAACGAAGCACCTTG	TCCGCCACTCAGTCACAATG
Bacteroides	GGTTCTGAGAGGAGGTCCC	CTGCCTCCCGTAGGAGT
Bacteroides fragilis	ATAGCCTTTCGAAAGRAAGAT	CCAGTATCAACTGCAATTTTA
Bifidobacterium spp.	ATTCTGGCTCAGGATGAACGC	CTGATAGGACGCGACCCCAT
Clostridia	CTCAACTTGGGTGCTGCATTT	ATTGTAGTACGTGTGTAGCCC
Clostridium cluster IV	GCACAAGCAGTGGAGT	CTTCCTCCGTTTTGTCAA
Clostridium coccoides	ACTCCTACGGGAGGCAGC	GCTTCTTAGTCAGGTACCGTCAT
Clostridium perfringens	CGCATAACGTTGAAAGATGG	CCTTGGTAGGCCGTTACCC
Clostridium subcluster XVIa	AAATGACGGTACCTGACTAA	CTTTGAGTTTCATTCTTGCGAA
Dorea	ACGGTACCTGACTAAGAAGCCC	CCTCAACGTCAGTCATCGTCC
E. coli	CATGCCGCGTGTATGAAGAA	CGGGTAACGTCAATGAGCAAA
Enterococcus spp.	CCCTTATTGTTAGTTGCCATCATT	ACTCGTTGTACTTCCCATTGT
Enterobacteriaceae	TGCCGTAACTTCGGGAGAAGGCA	TCAAGGCTCAATGTTCAGTGTC
EREC	ACTCCTACGGGAGGCAGC	GCTTCTTAGTCAGGTACCGTCA
Eubacteria(Universal)	ACTCCTACGGGAGGCAGCAGT	ATTACCGCGGCTGCTGGC
Firmicutes	GGAGYATGTGGTTTAATTCGAAGCA	AGCTGACGACAACCATGCAC
Flexispira	AATACATGCAAGTCGAACGATGA	AATCACCGTTTCCAGTGGCT
Helicobacter	CTTAACCATAGAACTGCATTTGAAACTAC	GGTCGCCTTCGCAATGAGTA
Lactobacillus	AGCAGTAGGGAATCTTCCA	CACCGCTACACATGGAG

MIB	CCAGCAGCCGCGGTAATA	CGCATTCCGCATACTTCTC
P. gingivalis	CTTGACTTCAGTGGCGGCAG	AGGGAAGACGGTTTTCACCA
Prevotellaceae	CCAGCCAAGTAGCGTGCA	TGGACCTTCCGTATTACC
γ-Proteobacteria	TAACGCTTGGGAATCTGCCTRTT	CATCTRTTAGCGCCAGGCCTTGC
SFB	AGGAGGAGTCTGCGGCACATTAGC	CGCATCCTTTACGCCCAGTTATTC
Staphylococcus spp.	TTTGGGCTACACACGTGCTACAATGGACAA	AACAACTTTATGGGATTTGCWTGA
Streptococcus spp.	AGATGGACCTGCGTTGT	GCTGCCTCCCGTAGGAGTCT
TM7	GCAACTCTTTACGCCCAGT	GAGAGGATGATCAGCCAG
